# Concurrent BMP7 and FGF9 signalling governs AP-1 function to promote self-renewal of nephron progenitor cells

**DOI:** 10.1038/ncomms10027

**Published:** 2015-12-04

**Authors:** Sree Deepthi Muthukrishnan, Xuehui Yang, Robert Friesel, Leif Oxburgh

**Affiliations:** 1 Center for Molecular Medicine, Maine Medical Center Research Institute, 81 Research Drive, Scarborough, Maine 04074, USA; 2 Graduate School of Biomedical Sciences and Engineering, University of Maine, Orono, Maine 04469, USA

## Abstract

Self-renewal of nephron progenitor cells (NPCs) is governed by BMP, FGF and WNT signalling. Mechanisms underlying cross-talk between these pathways at the molecular level are largely unknown. Here we delineate the pathway through which the proliferative BMP7 signal is transduced in NPCs in the mouse. BMP7 activates the MAPKs TAK1 and JNK to phosphorylate the transcription factor JUN, which in turn governs transcription of AP-1-element containing G1-phase cell cycle regulators such as *Myc* and *Ccnd1* to promote NPC proliferation. Conditional inactivation of *Tak1* or *Jun* in cap mesenchyme causes identical phenotypes characterized by premature depletion of NPCs. While JUN is regulated by BMP7, we find that its partner FOS is regulated by FGF9. We demonstrate that BMP7 and FGF9 coordinately regulate AP-1 transcription to promote G1-S cell cycle progression and NPC proliferation. Our findings identify a molecular mechanism explaining the important cooperation between two major NPC self-renewal pathways.

## Abstract (web summary)

The growth factors BMP and FGF both stimulate the self-renewal of nephron progenitor cells (NPCs), but how these signals overlap is unclear. Here in the mouse, Muthukrishnan *et al*. find BMP7 and FGF9 coordinately regulate AP-1 transcriptional activity, promoting G1-S cell cycle progression and NPC proliferation.

## Graphical abstract

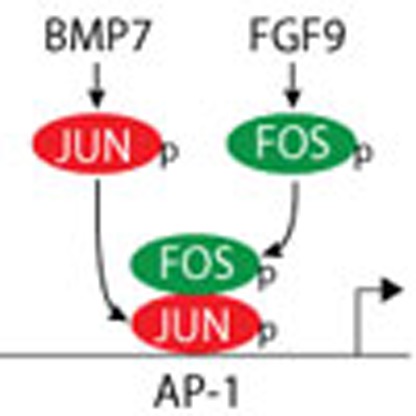

Iterative nephron induction is based on a program of reciprocal interactions between the ureteric bud and the surrounding nephron progenitor cells (NPCs) of the cap mesenchyme. The cap mesenchyme is divided into compartments expressing distinct transcription factors. The highest order compartment expresses Cbp/p300-interacting transactivator 1 (CITED1) and Six homeobox 2 (SIX2). This compartment transitions to the CITED1−/SIX2+ compartment that subsequently differentiates into the pre-tubular aggregate, the precursor of the epithelial renal vesicle^1^,^2^. A fine balance between NPC self-renewal and differentiation is critical in determining nephron number in the adult kidney. Three major growth factor pathways are essential for NPC maintenance and self-renewal: Wingless-type MMTV integration site family member 9B (WNT9B) /β-catenin, fibroblast growth factors (FGF) 9/20, and bone morphogenetic protein-7 (BMP7)/MAPK (refs 3, 4, 5, 6, 7). Previous reports suggest important signalling interactions in NPCs. For example, BMP and FGF synergistically promote progenitor cell maintenance in organ culture^8^. Understanding the mechanistic bases for these interactions is important to advance our understanding of renal organogenesis and for attempts at *de novo* nephrogenesis from stem cells.

To define the circuitry connecting signalling pathways and thus build an integrated model for regulation of NPC self-renewal, we first need to map signal transduction mechanisms used by each growth factor. In this study, we define the MAPK signalling cascade that transduces the proliferative response to BMP7 using complementary primary cell culture and conditional gene inactivation approaches. We show that the BMP7 signal is transduced through TAK1 and JNK to activate the transcription factor JUN in NPCs. JUN is required for proliferation of these cells and directly governs G1-phase cell cycle regulatory genes including *Myc* and *Ccnd1*. JUN is a component of the dimeric AP-1 transcription factor that also includes FOS (ref. 9). AP-1 components are differentially regulated by growth factor stimuli, and changes in dimer composition strongly influence AP-1 function^10^. Using both genetic and primary cell models we show that BMP7 and FGF9 have distinct effects on JUN and FOS. While FGF9 controls FOS activation, BMP7 activates JUN, which is the DNA-binding partner in the JUN-FOS heterodimer. JUN-FOS heterodimers are strong transactivators with enhanced DNA-binding ability compared with JUN homodimers^10^. Compared with either BMP7 or FGF9 treatment, combined BMP7 and FGF9 stimulation of NPCs amplifies activation of an AP-1 transcriptional reporter, increases transcription of the G1-S cell cycle regulator *Ccnd1*, and promotes G1-S transition and proliferation of cells. On the basis of these findings we propose that BMP7 and FGF9 cooperatively control AP-1 function to promote NPC proliferation, providing an explanation for the important synergy between these growth factors in NPC maintenance.

## Results

### BMP7 promotes NPC proliferation through TAK1 and JNK

**Figure 1:**
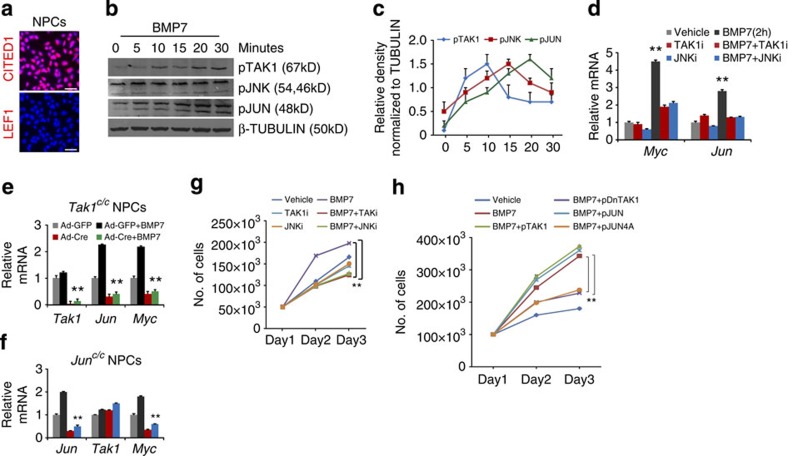
BMP7 activates TAK1-JNK-JUN signalling in NPCs. (**a**) CITED1 and LEF1 immunostaining of freshly purified E17.5 NPCs. Scale bars, 50 μM. (**b**) Immunoblot shows time course of activation of pTAK1, pJNK and pJUN in NPCs stimulated with BMP7 (Supplementary Fig. 1a). (**c**) Average protein densities measured at each time point from two independent experiments (*n*=2). Error bars represent s.d. (**d**) RT–qPCR of *Myc* and *Jun* on NPCs treated with vehicle, BMP7, TAK1 and JNK inhibitors for 2 h. Error bars represent s.d. **P<0.005, Student's *t*-test. Three biological replicates analysed per condition, *n*=3. (**e**,**f**) RT–qPCR of *Tak1*, *Jun* and *Myc* in *Tak1- and Jun*-inactivated NPCs stimulated with BMP7 for 2 h. Error bars represent s.d. **P<0.005, Student's *t*-test. Two biological replicates analysed per condition, *n*=2. (**g**) Growth curve of NPCs treated with vehicle, BMP7, TAK1 and JNK inhibitors. Average numbers are derived from five biological replicates per condition, *n*=5. Error bars represent s.d. **P<0.005, Student's *t*-test. (**h**) Growth curve of vehicle or BMP7-treated NPCs transfected with WT or kinase-dead constructs of TAK1 and JUN. Average numbers are derived from three biological replicates per condition, *n*=3. Error bars represent s.d. **P<0.009 and **P<0.005, Student's *t*-test.

Previous work from our laboratory indicated that BMP7 promotes proliferation of cells in the nephrogenic zone through MAPK signalling^7^. To determine the kinetics of pathway activation specifically in NPCs, we measured the phosphorylation states of each of the MAPK components TAK1, JNK and JUN in response to BMP7 in NPCs purified by immunomagnetic separation (Fig. 1a)^2^,^11^. Following BMP7 treatment, we detected a step-wise sequence of phosphorylation events with peak activation of pTAK1 at 10 min, pJNK at 15 min and pJUN at 20 min (Fig. 1b,c and Supplementary Fig. 1a). Activated JUN binds to AP-1 elements in target genes including itself and *Myc* (refs 12, 13). In NPCs, *Jun* and *Myc* were upregulated 2 h after BMP7 stimulation and pre-treatment with TAK1 and JNK inhibitors significantly reduced this response, indicating that they are early transcriptional targets of the pathway in these cells (Fig. 1d). *Tak1*- and *Jun*-deficient NPCs showed a significant reduction in BMP7 stimulation of *Jun* and *Myc* transcription, corroborating the finding that BMP7 controls transcription of *Jun* and *Myc* through TAK1-JNK signalling (Fig. 1e,f). *Myc* is required for renewal of NPCs *in vivo*, and our findings outline one signalling mechanism for the control of *Myc* expression in these cells^14^.

To evaluate the role of the BMP7-TAK1-JNK-JUN pathway in cellular proliferation, we assessed the growth curves of BMP7-stimulated NPCs treated with inhibitors of TAK1 or JNK. As expected, BMP7-stimulated proliferation was reversed by TAK1 or JNK inhibition (Fig. 1g). To confirm that NPCs retained their phenotype in the experimental conditions, we measured expression of CITED1, SIX2 and LEF1 as well as evaluating a panel of markers for cap mesenchyme and cortical interstitium (Supplementary Fig. 1b,c). To confirm that BMP7-stimulated proliferation depends on kinase activity of pathway components, wild type (WT) and kinase-dead versions of TAK1 and JUN were expressed in NPCs, which were stimulated with BMP7. Transfection efficiency was analysed by expressing a GFP construct, and by measuring the expression of *Tak1* and *Jun* transcripts in transfected NPCs (Supplementary Fig. 1d,e). Wild type TAK1 and JUN expression augmented the BMP7-induced proliferative response, whereas kinase-dead variants reduced it, confirming that phosphorylation of pathway components is essential for proliferation of NPCs (Fig. 1h and Supplementary Fig. 1f). On the basis of our primary cell analysis we conclude that BMP7 promotes NPC proliferation through activation of the TAK1-JNK-JUN signalling cascade.

### *Bmp7* and *Tak1* interact to control NPC renewal

**Figure 2:**
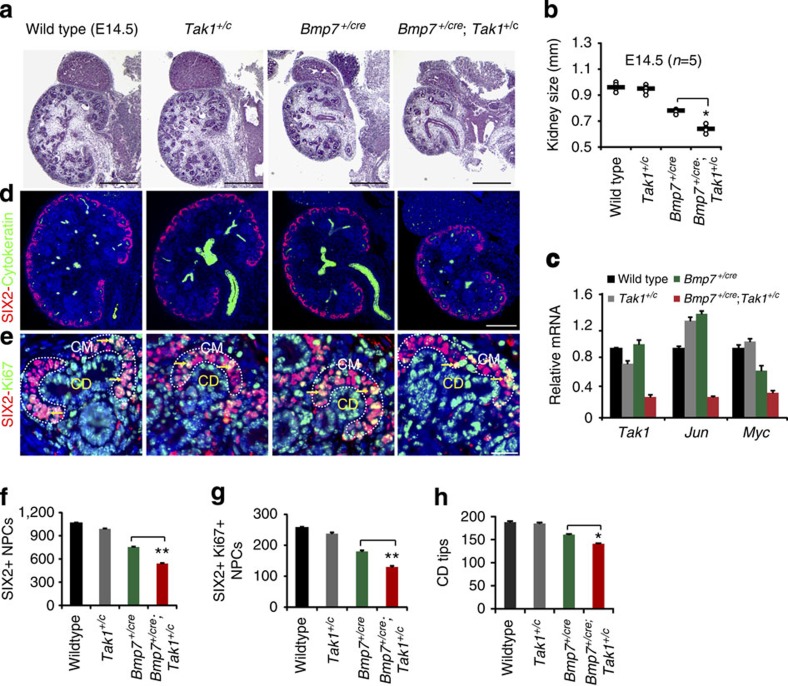
*Bmp7* and *Tak1* operate in the same pathway to regulate NPC renewal. (**a**) H&E staining of E14.5 WT, *Tak1*^*+/c*^, *Bmp7*^*+/cre*^ and *Bmp7*^*+/cre*^*;Tak1*^*+/c*^ kidneys. Scale bars, 500 μM. (**b**) Kidney sizes. Number of mice analysed per genotype is noted in the graph. *P<0.01, Student's *t*-test. (**c**) RT–qPCR of NPCs from P0 kidneys for *Tak1*, *Jun* and *Myc* in mutants and WT control. Error bars represent s.d. Two biological replicates analysed per genotype, *n*=2. (**d**) SIX2 (red, cap mesenchyme) and cytokeratin8 (green, collecting duct) immunostaining of whole kidney sections. Scale bars, 500 μM. (**e**) Co-immunostaining of SIX2 and the proliferation marker Ki67. Scale bars, 50 μM. (**f**–**h**) Quantitation of SIX2+, SIX2+/Ki67+, and collecting duct tips from 5 to 10 serial sections per kidney per genotype. Arrows point to Ki67+/SIX2+ cells in the cap mesenchyme, which is highlighted by white dashed lines. Error bars represent s.d. **P<0.005 and *P<0.05, Student's *t*-test. CD, collecting duct; CM, cap mesenchyme.

To confirm the BMP7-TAK1 relationship *in vivo*, we used the *Bmp7*^*+/cre*^ strain to inactivate a single copy of *Tak1*. *Bmp7*^*+/cre*^ is an inactivating mutation and heterozygous animals express only one copy of the gene^15^. We reasoned that limiting the availability of TAK1 would exacerbate the effect of reduced BMP7 ligand availability if these molecules operate in the same pathway. Although the body weight of *Bmp7*^*+/cre*^ embryos appeared slightly lower than WT, no difference could be detected between *Bmp7*^*+/cre*^ and *Bmp7*^*+/cre*^;*Tak1*^*+/c*^ mice (Supplementary Fig. 2a). Morphometric analyses at E14.5 and P0 revealed significant reductions in size (*P<0.05) and weight (**P<0.001, Student's *t*-test, *n*=6) of *Bmp7*^*+/cre*^;*Tak1*^*+/c*^ kidneys compared with *Bmp7*^*+/cre*^, supporting the notion that BMP7 indeed does signal through TAK1 *in vivo* (Fig. 2a,b and Supplementary Fig. 2b,c). To verify loss of *Tak1* and *Jun* in mutant kidneys, we measured expression of *Tak1*, *Jun*, and their downstream target *Myc* in isolated NPCs from P0 WT, *Tak1*^+/c^, *Bmp7*^*+/cre*^ and *Bmp7*^*+/cre*^;*Tak1*^*+/c*^ mice. *Tak1*, *Jun* and *Myc* were reduced by ∼60% in *Bmp7*^*+/cre*^;*Tak1*^*+/c*^ compared with WT (Fig. 2c). Activated pJUN levels were decreased in the cap mesenchyme, but not in the collecting duct tips of *Bmp7*^*+/cre*^;*Tak1*^*+/c*^ kidneys compared with *Bmp7*^*+/cre*^ and WT, confirming that compound heterozygosity for *Bmp7* and *Tak1* results in reduced activation of JNK-JUN signalling specifically in NPCs (Supplementary Fig. 2d,e).

Cell death in the nephrogenic zone and premature loss of NPCs are hallmarks of the *Bmp7* null mutants^16^,^17^,^18^. We therefore measured proliferation and cell death in NPCs of single and compound mutants. Using SIX2 with the proliferation marker Ki67, we observed a marked reduction in Ki67+/SIX2+ cells at E14.5 (15%) and P0 (10%), and a concomitant decrease in the number of SIX2+ cells per kidney in the *Bmp7*^*+/cre*^;*Tak1*^*+/c*^ kidneys relative to the *Bmp7*^*+/cre*^ mutant (Fig. 2d–g and Supplementary Fig. 2f–i). Apoptosis analysis showed no evidence of increased cell death in E14.5 *Bmp7*^*+/cre*^;*Tak1*^*+/c*^ or *Bmp7*^*+/cre*^ kidneys, suggesting that *Tak1* is involved only in the proliferative response of NPCs to BMP7 (Supplementary Fig. 2j). Growth and branching of the collecting duct is controlled by factors secreted by NPCs, and to determine if branching was secondarily affected in *Bmp7*^*+/cre*^;*Tak1*^*+/c*^ kidneys, we quantified the number of collecting duct tips. *Bmp7*^*+/cre*^ kidneys show reduced branching relative to the WT, which strongly suggests an effect of diminished NPC numbers in this mutant considering that the reduction of *Bmp7* caused by heterozygosity is predicted to promote ureteric bud outgrowth and branching^19^,^20^. Compared with *Bmp7*^*+/cre*^, *Bmp7*^*+/cre*^;*Tak1*^*+/c*^ kidneys showed a further reduction of collecting duct branching proportional to the reduction in NPC number (Fig. 2h and Supplementary Fig. 2k). Overall, our genetic interaction study supports control of NPC renewal by BMP7 signalling through TAK1 in the developing kidney.

### Deletion of *Tak1* and *Jun* in NPCs reduces their renewal

**Figure 3:**
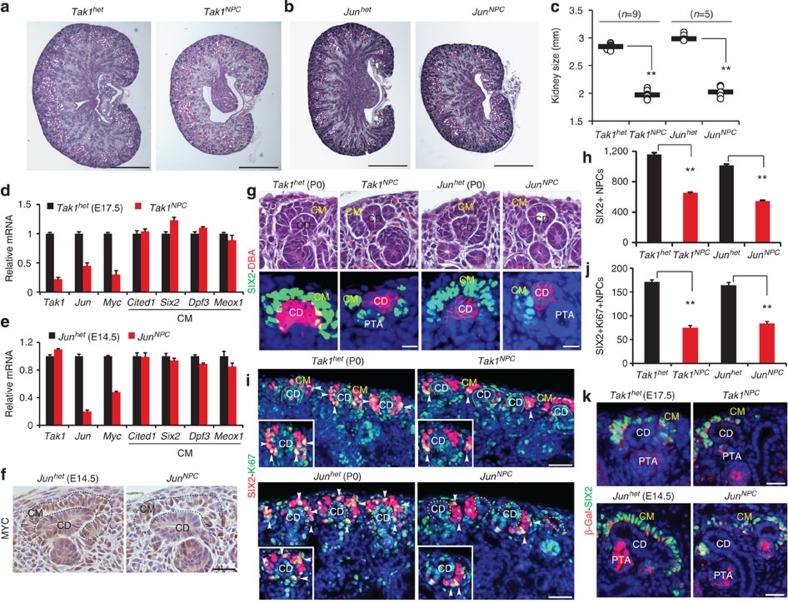
*Tak1* and *Jun* are required for self-renewal of NPCs throughout nephrogenesis. (**a**,**b**) H&E staining of P0 *Tak1*^*het*^, *Tak1*^*NPC*^, *Jun*^*het*^ and *Jun*^*NPC*^ kidneys. Scale bars, 500 μM (**c**) Kidney sizes. Number of mice (*n*) analysed per group is noted in the graph. **P<0.005, Student's *t*-test. (**d**,**e**) Transcriptional analysis of *Tak1*, *Jun*, *Myc* and cap mesenchyme markers in NPCs isolated from E17.5 *Tak1*^*het*^, *Tak1*^*NPC*^ and E14.5 *Jun*^*het*^, *Jun*^*NPC*^ kidneys. Error bars represent s.d. Two biological replicates analysed per genotype, *n*=2. (**f**) MYC immunostaining of E14.5 *Jun*^*het*^ and *Jun*^*NPC*^ kidneys. Scale bars, 50 μM. (**g**) H&E and co-immunostaining of SIX2 (green, cap mesenchyme) and DBA lectin (red, collecting duct) of P0 *Tak1*^*NPC*^ and *Jun*^*NPC*^ kidneys. Scale bars, 25 μM. (**h**) Average number of SIX2+ NPCs per kidney section per genotype. Error bars indicate s.d. **P<0.005, Student's *t*-test. (**i**) Ki67 (green) and SIX2 (red) co-immunostaining of P0 *Tak1*^*het*^, *Tak1*^*NPC*^, *Jun*^*het*^, *Jun*^*NPC*^ kidneys. Insets show magnifications of cap mesenchymes with arrows pointing to Ki67+ cells. Scale bars, 100 μM. (**j**) Number of SIX2+/Ki67+ cells per kidney section. Error bars represent s.d. and **P<0.005, Student's *t*-test (**k**) β-galactosidase (red) and SIX2 (green) co-immunostaining of E17.5 *Tak1*^*het*^ (*Six2*^*+/cre*^;*Tak1*^*+/c*^;*R26RLacZ*) and *Tak1*^*NPC*^ (*Six2*^*+/cre*^;*Tak1*^*c/c*^;*R26RLacZ*) kidneys. Three mice were analysed per genotype at E14.5 and E17.5 (*n*=3). CD, collecting duct; CM, cap mesenchyme; PTA, pre-tubular aggregate.

To stringently determine the requirement for components of the BMP7-TAK1-JNK-JUN pathway in NPCs *in vivo*, we inactivated *Tak1* and *Jun* using *Six2-cre*. Both *Tak1*^*NPC*^ (*Six2-cre*;*Tak1*^*c/c*^) and *Jun*^*NPC*^ (*Six2-cre*;*Jun*^*c/c*^) P0 kidneys showed significant reduction in kidney weight (50%) and size (30–40%) compared with *Tak1*^*het*^ (*Six2-cre;Tak1*^*+/c*^) and *Jun*^*het*^ (*Six2-cre;Jun*^*+/c*^) kidneys (**P<0.005, Student's *t*-test), confirming that these genes are essential in the *Six2* lineage, which is limited to NPCs and their derivatives (Fig. 3a–c and Supplementary Fig. 3a)^21^. Body weights of these different strains did not show significant differences (*P*>0.05, Student's *t*-test; Supplementary Fig. 3b). To verify loss of *Tak1* and *Jun*, we measured expression of *Tak1*, *Jun* and their target *Myc* in NPCs isolated at E17.5 (*Tak1*^*NPC*^ and *Tak1*^*het*^) or E14.5 (*Jun*^*NPC*^ and *Jun*^*het*^)*. Tak1* and *Jun* were reduced by 80% and *Myc* by ∼50–60% in *Tak1*^*NPC*^ and *Jun*^*NPC*^ NPCs, respectively (Fig. 3d,e). As expected, *Tak1* was unchanged in *Jun*^*NPC*^ NPCs (Fig. 3e). pJUN and MYC were reduced in mutant kidneys, confirming that inactivation of *Tak1* and *Jun* results in reduced activation of JNK-JUN signalling and downstream targets in NPCs (Fig. 3f and Supplementary Fig. 3c).

Morphologically, *Tak1*^*NPC*^ and *Jun*^*NPC*^ kidneys revealed several atypically organized cap mesenchymes carrying few NPCs (Fig. 3g and Supplementary Fig. 3d,e). SIX2+ cells were reduced by ∼50% in mutant kidneys, suggesting that *Tak1* and *Jun* inactivation results in premature loss of NPCs (Fig. 3h and Supplementary Fig. 3e). To understand if this was due to reduced proliferation, we measured coexpression of Ki67 or pHH3 and SIX2 in *Tak1*^*NPC*^ (P0) and *Jun*^*NPC*^ (E14.5 and P0) kidneys. We observed 50% reduction of Ki67+/SIX2+ cells and pHH3+/SIX2+ cells in *Tak1*^*NPC*^ (P0) and *Jun*^*NPC*^ (E14.5, P0) kidneys (Fig. 3i,j and Supplementary Fig. 3e). TUNEL and caspase3 staining showed no evidence of cell death in the *Tak1*^*NPC*^ kidneys, suggesting that loss of NPCs was strictly due to reduced proliferation (Supplementary Fig. 3f).

To rule out the possibility that *Tak1* and *Jun* mutant NPCs may take on a cortical interstitial fate, we analysed *Tak1*^*NPC*^ (E17.5) and *Jun*^*NPC*^ (E14.5) NPCs for markers of cap mesenchyme (*Cited1*, *Six2*, *Dpf3 and Meox1*) and cortical interstitium (*Foxd1* and *Sfrp1)*. Cap mesenchyme markers either remained unchanged or showed a slight increase in *Tak1*^*NPC*^ (E17.5) and *Jun*^*NPC*^ (E14.5). However, neither *Foxd1* nor *Sfrp1* were elevated in *Tak1*^*NPC*^ or *Jun*^*NPC*^ indicating that the cellular identity of NPCs is unaltered (Fig. 3d,e and Supplementary Fig. 3g). To confirm that *Tak1* and *Jun* mutant NPCs retain their cellular identity *in vivo*, we performed lineage tracing analysis by crossing the *Six2*^*+/cre*^*;Tak1*^*+/c*^ and *Six2*^*+/cre*^*;Jun*^*+/c*^ mice with the *R26RLacZ* reporter. β-galactosidase and SIX2 immunostaining revealed that tagged cells were confined to SIX2+ cap mesenchyme and its derivatives in both *Tak1*^*NPC*^ and *Jun*^*NPC*^ kidneys (Fig. 3k and Supplementary Fig. 3h). Thus, inactivation of *Tak1* and *Jun* in NPCs partially phenocopies the *Bmp7* null phenotype, suggesting that they operate in the same pathway to regulate NPC self-renewal.

**Figure 4:**
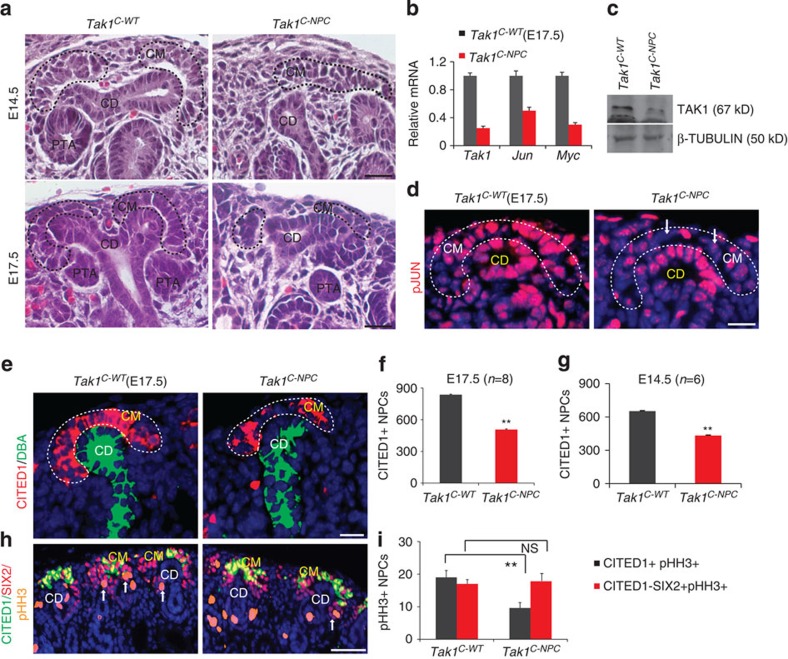
BMP7-mediated TAK1 signalling is required for proliferation of CITED1+ NPCs. (**a**) H&E staining of E14.5 and E17.5 *Tak1*^*C-WT*^ and *Tak1*^*C-NPC*^ kidneys shows nephrogenic units in which cap mesenchymes are outlined with black dashed lines. Scale bars, 50 μM (**b**) RT–qPCR of *Tak1*, *Jun* and *Myc* in NPCs isolated from E17.5 *Tak1*^*C-WT*^ and *Tak1*^*C-NPC*^ kidneys. Error bars indicate s.d. Two biological replicates analysed per genotype (*n*=2). (**c**) Immunoblot of TAK1 and β-Tubulin in NPCs isolated from E17.5 *Tak1*^*C-WT*^ and *Tak1*^*C-NPC*^ kidneys (Supplementary Fig. 4c). (**d**) pJUN immunostaining of E17.5 *Tak1*^*C-WT*^ and *Tak1*^*C-NPC*^ kidneys. Scale bars, 50 μM. (**e**–**g**) CITED1 (red) and DBA lectin (green) staining of E17.5 *Tak1*^*C-WT*^ and *Tak1*^*C-NPC*^ kidneys. Quantitation of numbers of CITED1+ cells per E14.5 and E17.5 kidney section per genotype. Number of mice (*n*) per genotype is noted in the graph. Error bars represent s.d. **P<0.005, Student's *t*-test. (**h**,**i**) CITED1 (green), SIX2 (red) and pHH3 (orange/proliferation marker) staining in E17.5 *Tak1*^*C-WT*^ and *Tak1*^*C-NPC*^. Scale bars, 150 μM. Quantitation of numbers of pHH3+/CITED1+/SIX2+ versus pHH3+/CITED1−/SIX2+ cells per kidney section per genotype. Error bars indicate s.d. **P<0.005 and *P*=0.08 (NS, not significant), Student's *t*-test. Cap mesenchymes highlighted with black or white dotted lines. CD, collecting duct; CM, cap mesenchyme; PTA, pre-tubular aggregate.

The few Ki67+/SIX2+ NPCs we observed in *Tak1*^*NPC*^ kidneys localized predominantly to the distal cap mesenchyme under the collecting duct tips, in which CITED1 expression is normally lost (see insets in Fig. 3i). To understand if the *Tak1*^*NPC*^ phenotype results from gene inactivation specifically in the CITED1+ compartment, we used the *Cited1-creER*^*T2*^ strain. *Cited1-creER*^*T2*^;*Tak1*^*c/c*^ (*Tak1*^*C-NPC*^) and *Tak1*^*c/*c^ (*Tak1*^*C-WT*^, littermate control) mice were tamoxifen injected at either E11.5 or E14.5 and collected after 72 h. *Tak1*^*C-NPC*^ kidneys were significantly smaller than *Tak1*^*C-WT*^ at both time points (**P<0.005, Student's *t*-test), and their cap mesenchymes were depleted (Fig. 4a and Supplementary Fig. 4a,b). *Tak1* transcript was reduced by 80% and both *Jun* and *Myc* were reduced in mutant NPCs (Fig. 4b). Immunoblotting confirmed the reduction of TAK1 in mutant NPCs (Fig. 4c and Supplementary Fig. 4c). pJUN and MYC were also reduced, indicating that *Tak1* inactivation results in reduced JNK-JUN signalling in NPCs (Fig. 4d and Supplementary Fig. 4d,e). To understand if *Tak1* is required to maintain NPCs in the CITED1+/SIX2+ state, we analysed cap mesenchyme markers in *Tak1*^*C-NPC*^ NPCs. *Tak1*-inactivated NPCs maintained expression of *Cited1* and *Six2* at levels similar to WT, indicating that they retain the appropriate cellular identity. However, the number of CITED1+ NPCs was reduced by 25–30% at both E14.5 and E17.5 (Fig. 4e–g and Supplementary Fig. 4f). Co-immunostaining for CITED1, SIX2 and pHH3 confirmed decreased pHH3 staining in CITED1+/SIX2+ cells of *Tak1*^*C-NPC*^ kidneys relative to *Tak1*^*C-WT*^ but no difference in the CITED1−/SIX2+ compartment at either E14.5 or E17.5, validating our findings from *Tak1*^*NPC*^ mutants (Fig. 4h,i and Supplementary Fig. 4g). Collectively, our genetic analysis suggests that the BMP7-TAK1-JNK-JUN pathway is required for proliferation of the early CITED1+/SIX2+ compartment *in vivo*.

### BMP7 promotes G1 to S cell cycle progression in NPCs

**Figure 5:**
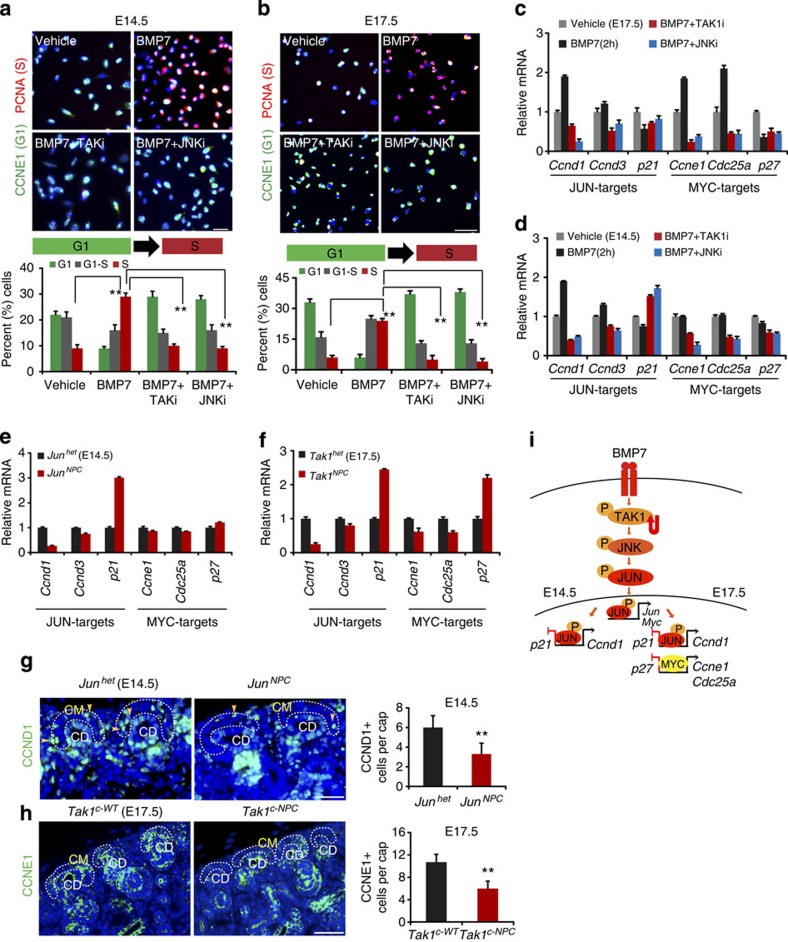
BMP7 promotes G1 to S cell cycle progression in NPCs. (**a**,**b**) CCNE1 (G1-phase) and PCNA (S-phase) immunostaining of E14.5 and E17.5 NPCs treated for 24 h with BMP7, TAK1 and JNK inhibitors. Graphs show the percentage of G1, G1-S and S-phase cells per condition. Three biological replicates analysed per condition (*n*=3). Error bars represent s.d. **P<0.005 and **P<0.001, Student's *t*-test. (**c**,**d**) RT–qPCR of JUN and MYC targets in E14.5 and E17.5 NPCs treated with vehicle, BMP7, TAK1 and JNK inhibitors for 2 h. Error bars indicate s.d. Three biological replicates analysed per condition (*n*=3). (**e**,**f**) RT–qPCR of JUN and MYC targets on NPCs isolated from E14.5 *Jun*^het^, *Jun*^*NP*C^ and E17.5 *Tak1*^het^, *Tak1*^*NPC*^ kidneys. Error bars represent s.d. Two biological replicates analysed per genotype (*n*=2). (**g**,**h**) CCND1 and CCNE1 immunostaining in E14.5 *Jun*^*het*^, *Jun*^*NPC*^ and E17.5 *Tak1*^*C-WT*^, *Tak1*^*C-NPC*^ kidneys. Scale bars, 100 and 150 μM. Quantitation of CCND1+ and CCNE1+ cells per cap mesenchyme (yellow arrows) was calculated by scoring at least 30 random cap mesenchymes per kidney section per genotype for a total of 5 sections from each experimental group. Error bars indicate mean (s.d.). **P<0.005 and **P<0.001, Student's *t*-test. (**i**) Model for BMP7-TAK1-JNK-JUN pathway regulation of NPC self-renewal in early (E14.5) and later (E17.5) phases of nephrogenesis. CD, collecting duct; CM, cap mesenchyme.

Having defined the requirements for components of the BMP7-TAK1-JNK-JUN signalling cascade in NPCs, we wanted to understand how this pathway interfaces with cellular proliferation control mechanisms. We have shown that the BMP7-TAK1-JNK-JUN pathway activates *Jun* and *Myc* transcription in NPCs (Figs 3 and 4). JUN and MYC are key transcriptional regulators of the cell cycle that modulate expression of genes involved in the G1-phase^22^,^23^. To test how the BMP7-TAK1-JNK-JUN pathway controls NPC proliferation, we investigated the effects of inhibiting pathway components on G1-S transition in BMP7-stimulated NPCs. Immunostaining for specific markers of G1 (CCNE1) and S (PCNA) was performed to calculate the percentages of G1 and S-phase cells in each experimental condition. After 24 h stimulation, BMP7 robustly promoted G1 to S transition in both E14.5 and E17.5 NPCs (Fig. 5a,b). TAK1 or JNK inhibition reversed this effect, confirming that BMP7 promotes NPC proliferation by controlling G1 to S transition through TAK1-JNK-JUN signalling (Fig. 5a,b and Supplementary Fig. 5a,b).

To understand the mechanism underlying the effect of BMP7-TAK1-JNK-JUN signalling on the G1-S transition, we set out to define the repertoire of G1-phase cell cycle regulatory genes modulated by the pathway in NPCs. Several cell cycle regulators containing AP-1-binding sites are JUN targets including *Ccnd1*, *Ccnd3*, *p21*, *p16*, *Jun* and *Myc* (refs 24, 25). Like JUN, MYC regulates the cell cycle by controlling G1-phase genes. Although a number of targets are shared, MYC has a unique repertoire including *Ccne1*, *Cdc25a*, *p27* and *Ccna2* (ref. 22). BMP7 may therefore control G1-S cell cycle regulators not only through JUN but also through MYC. Conditional gene inactivation shows that *Myc* is required for NPC renewal at E15.5-E18.5 but not earlier in nephrogenesis, suggesting that the contribution of MYC to cell cycle control by BMP7 might be limited to later stages of nephrogenesis^14^. To understand if this is the case, we compared the responsiveness of MYC and JUN targets to BMP7 in NPCs at E14.5 and E17.5. The JUN targets *Ccnd1*, *Ccnd3* and *p21* were regulated by BMP7 in a TAK1- and JNK-dependent manner in both E14.5 and E17.5 NPCs (Fig. 5c,d). However, the MYC targets *Ccne1*, *Cdc25a* and *p27* were regulated by BMP7 in a TAK1- and JNK-dependent manner only in E17.5 NPCs (Fig. 5c,d and Supplementary Fig. 5c–f). Our analysis indicates that the BMP7-TAK1-JNK-JUN pathway regulates JUN cell cycle targets including *Myc* throughout nephrogenesis, but that the contribution of MYC itself to control of G1 targets is limited to later stages of nephrogenesis.

To confirm these observations *in vivo*, we first measured target gene activation in NPCs isolated from E14.5 *Jun*^*NPC*^ and E17.5 *Tak1*^*NPC*^ kidneys. As expected, JUN targets were misregulated in mutant NPCs at both E14.5 and E17.5, whereas MYC targets were misregulated only at E17.5 (Fig. 5e,f). Next, we immunostained *Bmp7* null, *Jun*^*NPC*^ and *Tak1*^*C-NPC*^ kidneys for the JUN-activated target CCND1 and the MYC-activated target CCNE1 at E14.5 and E17.5. CCND1 has been used as a marker of the distal tubule; therefore we first verified its expression in cap mesenchyme using two different antibodies^26^. CCND1 was expressed in a salt-and-pepper distribution in WT cap mesenchyme, as expected considering that its expression is limited to the G1-phase of the cell cycle (Fig. 5g and Supplementary Fig. 5g). CCND1 expression was reduced in the cap mesenchymes of all mutants, suggesting that the BMP7-TAK1-JNK-JUN pathway indeed controls CCND1 *in vivo* and regulates JUN targets both early and late in nephrogenesis (Fig. 5g and Supplementary Fig. 5i). To understand if this could represent a general reduction in expression of G1 cell cycle genes in NPCs of mutant kidneys, we also measured expression of *Ccnd3*, which is expressed in a temporally overlapping manner with *Ccnd1*. Although RNA expression was reduced by 20%, protein expression was not significantly altered in mutants, supporting the notion that CCND1 is specifically misregulated in BMP7-TAK1-JNK-JUN pathway mutants *in vivo* (Supplementary Fig. 6h). Expression of the MYC-activated target CCNE1 was reduced in the E17.5 mutant kidneys but not in the E14.5 mutants, confirming our previous observation that MYC targets are regulated by the BMP7-TAK1-JNK-JUN pathway preferentially at later stages of nephrogenesis (Fig. 5h and Supplementary Fig. 6j). From these analyses, we conclude that the BMP7-TAK1-JNK-JUN pathway controls cellular proliferation of NPCs by regulating different G1-phase cell cycle regulators in early and later phases of nephrogenesis (Fig. 5i).

### BMP7 and FGF9 cooperatively control AP-1 transcription

**Figure 6:**
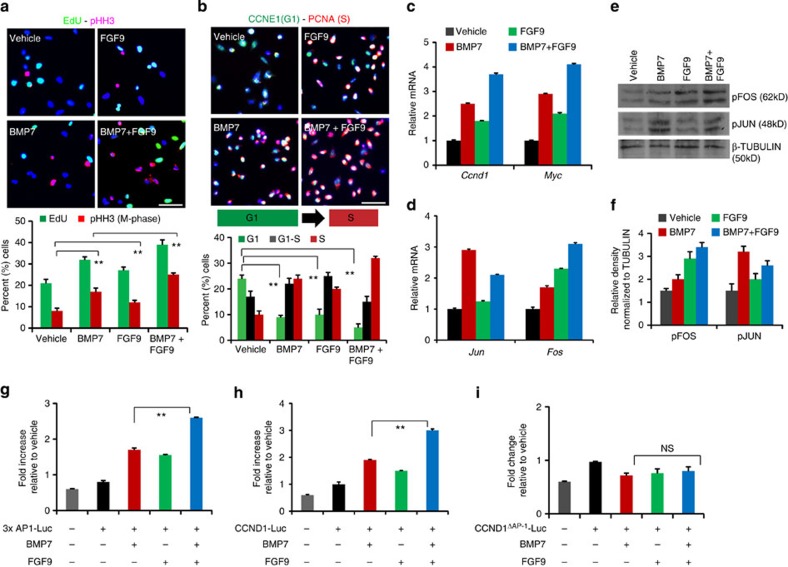
Combinatorial BMP7 and FGF9 signalling controls AP-1 function in NPCs. (**a**) Immunostaining of EdU (S-phase) and pHH3 (M-phase), (**b**) CCNE1 (G1-phase) and PCNA (S-phase) in E17.5 NPCs stimulated with vehicle, BMP7, FGF9 or BMP7+FGF9 for 24 h. Scale bars, 50 μM. Graphs show the percentage of EdU+,pHH3+ cells and G1, G1-S and S-phase cells in each condition. Error bars represent mean (s.d.). **P<0.005 and *P*<0.001 (Student's *t*-test). Two to three biological replicates analysed per condition (*n*=2 in **a**) and (*n*=3 in **b**) (**c**,**d**) RT–qPCR of cell cycle genes (*Ccnd1* and *Myc*), *Jun* and *Fos* in NPCs stimulated with vehicle, BMP7, FGF9 or BMP7+FGF9 for 2 h. Error bars represent s.d. Three biological replicates analysed per condition, *n*=3. (**e**,**f**) pJUN and pFOS immunoblot of NPCs stimulated with vehicle, BMP7, FGF9 or BMP7+FGF9 for 20 min. Graph shows the relative density of pJUN and pFOS normalized to β-tubulin in each condition (Supplementary Fig. 6e). (**g**–**i**) Bars in the graphs represent the average fold change in luciferase activity of 3xAP1-Luc, CCND1-Luc and CCND1^ΔAP-1^-Luc in NPCs stimulated with BMP7 and FGF9 relative to vehicle treatment for 24 h. Three biological replicates analysed per condition (*n*=3). Error bars represent s.d. **P<0.005, NS, not significant *P*>0.05, Student's *t*-test.

FGF9 has been reported to synergize with BMP7 to promote maintenance of isolated metanephric mesenchyme *in vitro*, however the molecular mechanism underlying this cross-talk remains unknown^5^,^8^. Metanephric mesenchyme consists of a mixture of cell types, and we first analysed proliferation in BMP7- and FGF9-treated purified E17.5 NPCs to understand if the pathways intersect in this cell type. Using 5′-ethynyl-2′deoxy-uridine (EdU) to label the S-phase and pHH3 to mark cells undergoing mitosis (M), we measured the overall proliferation of NPCs stimulated with BMP7, FGF9 or BMP7+FGF9. BMP7 or FGF9 stimulation showed a significant increase in EdU+ and pHH3+ nuclei compared with vehicle, and this effect was further augmented in BMP7+FGF9 stimulated cultures (Fig. 6a). Immunostaining and transcriptional analysis of cap mesenchyme markers showed that BMP7+FGF9-treated cultures remained in the CITED1+ state following treatments (Supplementary Fig. 6a,b). Quantitation of number of EdU+ and pHH3+ nuclei revealed a significant increase in S and M-phase cells in BMP7+FGF9-treated cultures relative to either BMP7 or FGF9 stimulation, suggesting that these growth factors indeed collaboratively promote NPC proliferation (Fig. 6a). To understand if FGF9 interfaces with BMP7 to regulate G1-S progression, we labelled NPCs treated with BMP7, FGF9, or BMP7+FGF9 with CCNE1 and PCNA to distinguish cells in G1 and S phases. BMP7+FGF9 stimulation resulted in ∼50% fewer G1 and G1-S cells and 30% more S-phase cells compared with BMP7 or FGF9 treatment suggesting that BMP7 and FGF9 promote NPC proliferation by accelerating the G1 to S cell cycle progression (Fig. 6b). To determine how FGF9 and BMP7 control the G1-S transition, we measured stimulation of the BMP7-TAK1-JNK-JUN-controlled G1 regulatory genes *Ccnd1*, *Ccnd3*, *Myc*, *Ccne1* and *Cdc25a* by either factor or both factors together. Expression of all five transcripts was upregulated by BMP7 and interestingly, FGF9 stimulation also increased their transcription indicating that FGF9 contributes to regulation of AP-1 targets. BMP7+FGF9 combinatorial stimulation showed an additive effect on these targets, indicating that BMP7 and FGF9 coordinately control transcription of G1-phase cell cycle regulators (Fig. 6c and Supplementary Fig. 6c).

AP-1 function is regulated by dimer composition as well as the phosphorylation status of its constituents, and JUN-FOS heterodimers activate targets more efficiently than JUN homodimers^9^,^10^. Given that BMP7 and FGF9 combinatorial stimulation increased transcription of G1-phase cell cycle regulators containing AP-1-binding elements, we speculated that FGF9 may modulate transcription and phosphorylation of JUN or its dimeric partner FOS concomitantly with BMP7 to regulate AP-1 function. We first measured the effects of FGF9 and BMP7 stimulation on *Jun* and *Fos* transcription. As expected, *Jun* transcription was upregulated by BMP7, but surprisingly it was unaffected by FGF9 (Fig. 6d). FGF9 stimulation of cells was verified by measuring expression of the FGF-target gene *Spry1* (Supplementary Fig. 6d). *Fos* transcription, on the other hand, was strongly induced by FGF9 compared to BMP7 and this effect was further enhanced by combined stimulation with BMP7. Thus, while FGF9 and BMP7 cooperatively promote *Fos* transcription, the obligate DNA-binding partner *Jun* is controlled by BMP7 alone (Fig. 6d). Examination of JUN and FOS phosphorylation showed that BMP7 robustly activates JUN (3.15-fold), whereas FGF9 activates FOS (2.85-fold) (Fig. 6e and Supplementary Fig. 6e). BMP7+FGF9 stimulation resulted in simultaneous phosphorylation of FOS and JUN suggesting that AP-1 transcriptional activation may be potentiated (Fig. 6f). To test this, we transfected NPCs with an AP-1 luciferase reporter (3 × AP1-Luc) and measured reporter activity in response to BMP7 and FGF9 stimulation^27^. BMP7 or FGF9 treatment resulted in less than a twofold luciferase response, but combined treatment caused more than a 2.5-fold increase, indicating that simultaneous JUN and FOS activation promotes AP-1 transcriptional activity (Fig. 6g). To test this in a gene that directly influences proliferation in NPCs, we compared activation of a *Ccnd1* luciferase reporter (CCND1-Luc) with a variant in which the AP-1-binding site has been mutated (CCND1^ΔAP-1^-Luc)^28^. BMP7 or FGF9 treatment alone showed less than twofold luciferase response, whereas BMP7+FGF9 stimulation resulted in a threefold increase, demonstrating that concurrent BMP7 and FGF9 signalling strongly promotes AP-1 function compared with either factor alone (Fig. 6h). Dependence on the AP-1 element for this transcriptional activity was confirmed by the finding that the *Ccnd1* promoter with mutated AP-1-binding site was unresponsive to BMP7 and/or FGF9 stimulation (Fig. 6i).

**Figure 7:**
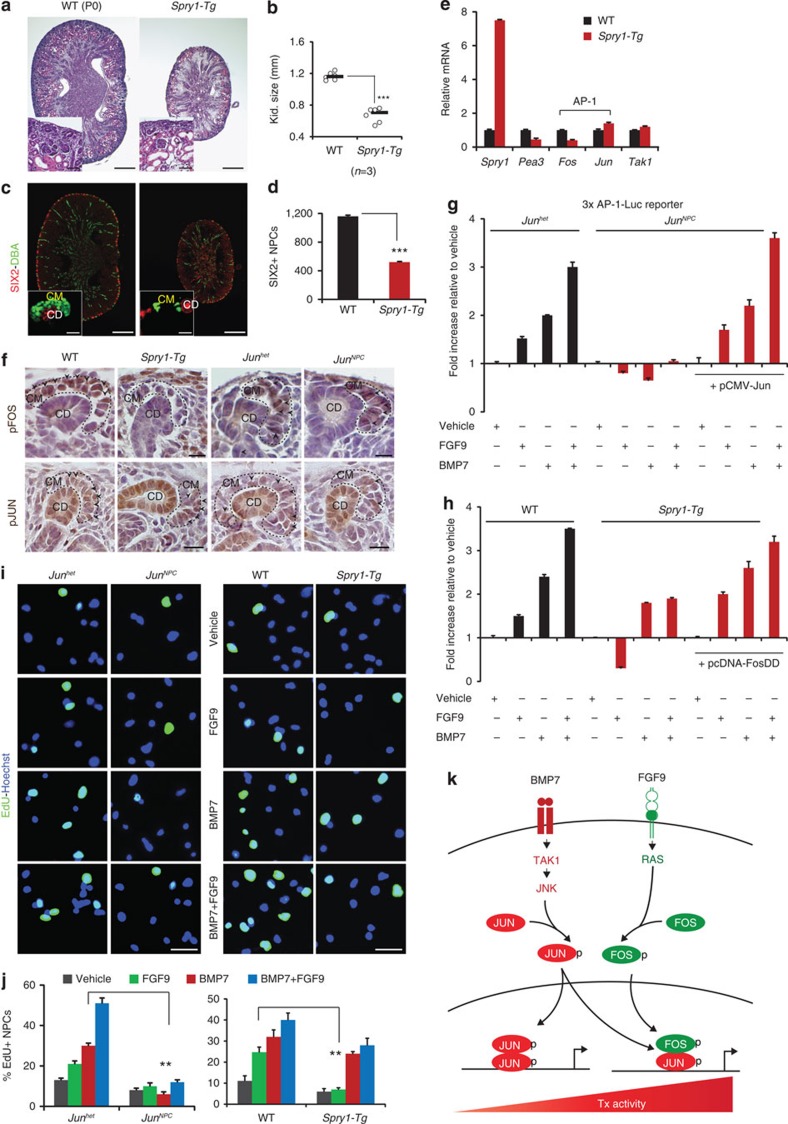
*Jun* is essential for AP-1 activation by BMP7 and FGF9 in NPCs. (**a**) H&E staining of P0 WT (*Six2-cre* or *Spry1-Tg* littermate controls) and *Spry1-Tg* (*Six2-cre*;*Spry1-Tg*) kidneys. Insets show higher magnification images of the nephrogenic zone. Scale bars, 500 and 50 μM. (**b**) Kidney size of P0 WT and *Spry1-Tg* kidneys. ***P<0.0005, Student's *t*-test. Number of mice (*n*) per genotype is noted in the graph. (**c**) SIX2 (red, cap mesenchyme) and DBA lectin (green, collecting duct) immunostaining of *Spry1-Tg* kidneys. Scale bars, 500 and 25 μM (**d**) Number of SIX2+ NPCs per section in P0 WT and *Spry1-Tg* kidneys. Error bars represent s.d. ***P<0.0005, Student' *t*-test. (**e**) RT–qPCR of *Spry1*, *Pea3* (FGF-target), *Fos* and *Jun* (AP-1 components) and *Tak1* in E17.5 WT and *Spry1-Tg* NPCs. Two biological replicates analysed per genotype (*n*=2). Error bars represent s.d. (**f**) Immunostaining of pFOS and pJUN (black arrows) on P0 WT, *Spry1-Tg* and *Jun*^*het*^, *Jun*^*NPC*^ kidneys. Scale bars, 50 μM. (**g**,**h**) Luciferase activity relative to vehicle treatment in E17.5 *Jun*^*het*^, *Jun*^*NPC*^ and WT, *Spry1-Tg* NPCs transfected with 3 × AP1-Luc and pCMV-JUN or pcDNA-FOSDD and treated with BMP7, FGF9 and BMP7+FGF9 for 24 h. Error bars represent s.d. Three biological replicates analysed per condition (*n*=3). (**i**,**j**) EdU labelling (green, proliferation marker) of E17.5 NPCs isolated from *Jun*^*het*^, *Jun*^*NPC*^ and WT and *Spry1-Tg* kidneys and treated with vehicle, BMP7, FGF9 and BMP7+FGF9 for 24 h. Average number of EdU+ NPCs scored in each condition. Two biological replicates analysed per condition (*n*=2). Error bars represent s.d. **P<0.005, Student's *t*-test. Scale bars, 50 μM. (**k**) Model for BMP7 and FGF9 collaborative regulation of NPC self-renewal. CD, collecting duct; CM, cap mesenchyme.

We previously reported that transgenic expression of the FGF feedback regulator *Spry1* in NPCs results in increased apoptosis in cap mesenchyme^6^,^29^. To confirm the contribution of FGF signalling to FOS regulation *in vivo*, we generated *Six2-cre*;*Spry1-Tg* mice. Kidneys were severely hypoplastic at P0, and body weight was reduced compared with WT littermate controls (Fig. 7a,b; and Supplementary Fig. 7a–c). *Spry1-Tg* kidneys revealed a thin nephrogenic zone with depleted cap mesenchymes and distended tubules (insets in Fig. 7a and Supplementary Fig. 7d). SIX2 immunostaining confirmed premature loss of NPCs in *Spry1-Tg* kidneys with ∼65% reduction in NPC number (Fig. 7d and Supplementary Fig. 7e). *Spry1* expression increased sevenfold in *Spry1-Tg* NPCs, whereas the FGF-target gene *Pea3* was reduced by 55%, confirming inhibition of FGF signalling. *Fos* transcript diminished by 70%, whereas *Jun* and its upstream regulator *Tak1* remained unchanged (Fig. 7e). To determine if *Spry1*-mediated attenuation of FGF signalling strictly results in reduced activation of FOS, we compared pFOS and pJUN expression in cap mesenchymes of P0 WT and *Spry1-Tg* versus *Jun*^*het*^ and *Jun*^*NPC*^ kidneys. Expression of pFOS was reduced, whereas pJUN levels remained intact in cap mesenchymes of *Spry1-Tg* kidneys. Reciprocally, pFOS levels were unperturbed while pJUN was strongly reduced in cap mesenchymes of *Jun*^*NPC*^ kidneys (Fig. 7f and Supplementary Fig. 7f). We next asked if reduced activation of JUN and FOS in *Jun*^*NPC*^ and *Spry1-Tg* NPCs, respectively, decreases AP-1 transcriptional activity in response to BMP7 or FGF9 stimulation, and whether this effect can be rescued by expressing JUN or FOS in mutant NPCs. We treated 3 × AP-1Luc-transfected E17.5 NPCs isolated from *Jun*^*het*^ and *Jun*^*NPC*^, WT and *Spry1-Tg* kidneys with BMP7 and/or FGF9. Interestingly, *Jun*-deficient NPCs failed to activate the AP-1 reporter in response to both BMP7 and FGF9 treatment, whereas *Spry1*-*Tg* NPCs only showed a slight reduction in AP-1 reporter activity in response to BMP7, but were completely unresponsive to FGF9 (Fig. 7g,h). Expression of a wild type JUN construct (pCMV-JUN) rescued AP-1 reporter activation in both BMP7- and FGF9-stimulated *Jun*-deficient NPCs, and expression of a FOS phosphorylation-mimic construct (pcDNA-FOSDD) rescued AP-1 reporter activity in FGF9 stimulated *Spry1*-*Tg* NPCs (Fig. 7g,h)^30^,^31^. This suggests that JUN is essential for AP-1 activation by both growth factors, and the availability of FOS determines the amplitude of AP-1 activity. Cellular identity and transfection efficiency of NPCs was verified by examining cap mesenchyme and pre-tubular aggregate markers, expression of a GFP construct, JUN and pJUN immunostaining and RT–qPCR analysis of *Jun* transcript levels in transfected cells (Supplementary Fig. 7g–l). To determine whether JUN and FOS are required for the proliferative response of NPCs to BMP7 and FGF9, we performed EdU labelling of NPCs isolated from E17.5 *Jun*^*het*^ and *Jun*^*NPC*^, WT and *Spry1-Tg* kidneys and stimulated with BMP7 and/or FGF9. *Jun*-deficient NPCs failed to respond to both BMP7 and FGF9 stimulation. However, proliferation of *Spry1*-*Tg* NPCs in response to FGF9 was severely attenuated, and only slightly reduced in response to BMP7 suggesting that *Jun* is essential for proliferative response of NPCs to both BMP7 and FGF9 (Fig. 7i,j).

On the basis of these studies, we propose that BMP7 and FGF9 cooperatively control the composition of AP-1 dimers in NPCs, and that AP-1 composition influences the strength of activation of cell cycle regulators such as *Ccnd1* (Fig. 7k).

## Discussion

BMP7 is required for NPC maintenance in the developing kidney^15^,^16^,^17^. We show that the signalling cascade is initiated by activation of the MAPKKK TAK1 in response to BMP7 stimulation. TAK1 can be activated by numerous stimuli, but the finding that *Bmp7* and *Tak1* interact to regulate NPC renewal *in vivo* indicates an essential role for TAK1 specifically in the BMP7 pathway^32^. TAK1 activates JNK, which phosphorylates the transcription factor JUN, and the kinase activity of each of these components is essential for proliferation of NPCs. Kidneys lacking *Tak1* or *Jun* in cap mesenchyme display identical phenotypes characterized by premature depletion of NPCs, indicating that JUN may be the sole essential mediator downstream of TAK1 in this signalling process. We show that *Myc* is a transcriptional target of the BMP7-TAK1-JNK-JUN pathway in NPCs. MYC is essential for proliferation of cap mesenchyme *in vivo* and, together with JUN, activates G1-phase cell cycle regulators, explaining the proliferative effect of BMP7 stimulation^14^. NPCs display different average cell cycle lengths during early (E13.5) and later (E17.5) stages of nephrogenesis. Proliferation profiles of cap mesenchymes at these stages suggest that they are heterogeneous, containing both slowly and rapidly dividing cells^33^. An interpretation consistent with models of other stem/progenitor cell populations is that the slowly dividing subset may represent the self-renewing CITED1+/SIX2+ population, whereas the rapidly dividing subset represents the CITED1−/SIX2+ population that is differentiating^34^,^35^. Our finding that the CITED1+/SIX2+ population is reduced following conditional inactivation of *Tak1* using *Six2-cre* and *Cited1-creER*^*T2*^ drivers without any notable effect on proliferation of the CITED1−/SIX2+ population indicates that the BMP7-TAK1-JNK-JUN pathway is used primarily by the slowly dividing, self-renewing cells in the cap mesenchyme. The cycle length of NPCs increases as the embryo ages indicating that control mechanisms are added as development progresses. Interestingly, MYC targets are primarily regulated late in nephrogenesis, which is consistent with the observation that conditional *Myc* inactivation slows cap mesenchyme proliferation only after E15.5. Whether cell cycle control through MYC late in nephrogenesis represents addition of a control mechanism or simply redundancy with N-MYC, whose expression is lost late in nephrogenesis will need to be answered by comparison of conditional inactivation of both factors^36^.

In addition to reduced proliferation in the cap mesenchyme, loss of *Bmp7* causes ectopic cell death within the nephrogenic zone^16^,^17^,^18^. We do not see any effects on survival in kidneys in which *Tak1* or *Jun* have been inactivated in the cap mesenchyme. However, inactivation of *Smad4* using *Bmp7*-*cre* results in ectopic cell death within the nephrogenic zone of mutant kidneys, indicating that cell survival may be regulated through the SMAD pathway^15^. Mechanisms that control the balance between SMAD versus TAK1 signalling downstream of BMP7 in the cap mesenchyme are not currently understood. However, recent work indicates that FGF signalling may negatively regulate SMAD signalling providing an explanation for the lack of SMAD signalling seen in the CITED1+ compartment^37^.

Composition of the AP-1 dimer is a critical factor in determining cellular fates such as proliferation, apoptosis and differentiation^10^. We find that BMP7 robustly controls transcription and activation of JUN, whereas FGF9 strongly induces transcription and phosphorylation of FOS. Analysis of the *Six2-cre*;*Spry1-Tg* mouse strain in which the FGF feedback inhibitor SPRY1 is expressed in NPCs and primary cell transcriptional reporter assays indicates that the activation of JUN and FOS by simultaneous BMP7 and FGF9 signalling potentiates AP-1 transcription, and we propose that co-regulation of the AP-1 transcription factor is one basis for the cooperative effect of BMP7 and FGF9 in kidney development. While we have defined the signalling cascade between BMP7 and *Jun*, the pathway between FGF9 and *Fos* is less clear. The inhibitory effect of *Spry1* indicates that RAS is essential, and a recent report showing that NPC self-renewal is dependent on PI3K suggests that FGF9-RAS-PI3K may be the pathway governing *Fos* expression^38^.

Although JUN can form homodimers to activate target transcription, these bind AP-1 elements less tightly than JUN-FOS heterodimers, and have weaker transcriptional activity. In the case of BMP7 stimulation alone, the ratio would be skewed towards homodimer formation, whereas concurrent FGF9 signalling would skew the ratio towards JUN-FOS heterodimers, thus amplifying target transcription. Combinatorial BMP7 and FGF9 stimulation promotes robust transcription of the G1 regulator *Ccnd1* in an AP-1-dependent manner, and we propose that control of AP-1 targets by combinatorial BMP7 and FGF9 signalling promotes G1-S transition, explaining a mechanism for the cooperative effects of these growth factors on NPC proliferation.

Our current findings identify AP-1 as a specific point of interaction of the BMP and FGF pathways in NPCs. We have previously shown that WNT and FGF activate common targets in NPCs^6^. It therefore seems possible that WNT9B-β-catenin signalling could converge with BMP7 and FGF9 on the regulation of AP-1. Signalling cross-talk between BMP, FGF and WNT pathways is a recurring theme in organogenesis, and WNT/β-catenin signalling can regulate transcription of AP-1 targets such as *Myc, Ccnd1* and *Ccnd2* (refs 39, 40). Understanding this point of intersection further could explain the molecular basis for the combinatorial effects of these three distinct pathways on NPC proliferation.

## Methods

### Mouse strains

Animal care was in accordance with the National Research Council Guide for the Care and use of laboratory animals and protocols were approved by the Institutional Animal Care and Use Committee of Maine Medical Center. *Cited1-creER*^*T2*^ mice, *R26RlacZ* mice and *Spry1-Tg* mice were maintained on an FVB/NJ background^29^,^41^,^42^. *Bmp7*^*+/cre*^ mice were maintained on an ICR background*. Six2-TGC*^*tg*^ (*Tg(Six2-EGFP/cre)*^*1Amc/J*^), *Tak1*^*c/c*^ (*Map3k7*^*tm1.1Mds*^), *Tak1*^*+/c*^, and *Jun*^*c/c*^ (*Jun*^*tm4wag*^) mice were maintained on the C57BL/6 background^21^,^43^,^44^. For tamoxifen-inducible cre mice, pregnant dams were injected at the indicated times with 3 mg tamoxifen in corn oil per 40 g mouse.

### Plasmid constructs and adenoviral vector transduction

WT and kinase-defective TAK1 (K63W) plasmids were gifts from Dr Jun Ninomiya-Tsuji^37^. pCX-EGFP construct was a gift from Dr Andras Nagy^45^. FLAG-JUNWT-Myc and FLAG-JUN4A-MYC were gifts from Dr Axel Behrens (Addgene # 47443, 47444)^30^. FOS-DD was a gift from Dr John Blenis (Addgene # 8698)^31^. 3 × AP1-pGL3 was a gift from Dr Alexander Dent (Addgene # 40342)^27^. pGL3Basic-962 CCND1 promoter luciferase), pGL3Basic-962 CCND1 promoter AP-1 site mutant (Addgene #32727 and # 32728) were gifts from Dr Frank McCormick^28^. pRL-CMV (Renilla-Luciferase) was obtained from Promega. Adenoviral vectors (Ad-Cre and Ad-GFP) transduction was performed as described previously with a multiplicity of infection (MOI) of 500 for 40 h under serum-free conditions^7^.

### Immunohistochemistry

Dissected kidneys were fixed in 4% paraformaldehyde (PFA) for 30 min (E14.5, E17.5 kidneys) to 1 h (P0 kidneys) at room temperature. Paraffin-embedded sections were incubated with blocking solution containing 1X phosphate buffered saline (PBS), 1% bovine serum albumin, 5% serum of secondary antibody species (Jackson ImmunoResearch) and 0.05% hydrogen peroxide (Sigma) for 1 h at room temperature. Primary antibodies were diluted in 1X PBS and incubated at 4 °C overnight: anti-SIX2 (1:200–1:400, Proteintech), anti-CITED1 (1:200, Neomarkers), anti-GFP (1:200, Abcam), anti-LEF1 (1:100, Cell signaling), anti-pHH3 (1:100, Cell signaling), anti-Ki67 (1:200, Developmental Studies Hybridoma Bank (DSHB)), anti-caspase3 (1:100, Cell signaling), anti-Cytokeratin8/TROMA-I (1:200, DSHB), anti-DBA Lectin (1:500, Vector Laboratories), anti-JUN and anti-pJUN (1:200, Cell signaling), anti-FOS and anti-pFOS (1:400, Cell signaling), anti-CCND1 (1:100, Cell signaling), anti-CCND3 (1:100, Cell signaling), anti-MYC (1:100, Abcam), anti-CCNE1 (1:250, Santacruz) and anti-PCNA (1:200, Santacruz). Alexa-Fluor 488/568/647 conjugated secondary antibodies were used at 1:250 for detection of labelled cells. Nuclei were stained using DAPI (Molecular probes) for immunofluorescence and hematoxylin for immunohistochemistry. Sections were mounted using vectashield Mounting medium (Vector Laboratories). TUNEL staining was performed using ApopTagPlus peroxidase *in situ* apoptosis detection kit (EMD Millipore) according to manufacturer's instructions.

### Purification of NPCs by magnetic bead depletion

Total NZCs were isolated from E14.5 and E17.5 ICR mice by enzymatic digestion as previously described^7^. For isolation of NZCs from E17.5 and P0 conditional mutants, control and mutant kidneys were sorted based on size and GFP expression (*Six2-cre-EGFP* and *Cited1-creER*^*T2*^*-EGFP*), and confirmed by genotyping. Enrichment for CITED1+ cells and purification (referred to as NPCs) was performed by negative depletion with magnetic activated cell sorting using phycoerythrin-conjugated antibodies and anti-phycoerythrin Microbeads according to the manufacturer's protocol (Miltenyi Biotec)^2^,^11^. Purified NPCs were cultured in monolayer in keratinocyte serum-free media (KSFM, Thermo Fisher Scientific) supplemented with rh-FGF2 (50 ng ml^−1^, R&D Systems) and 100 U ml^−1^ penicillin–streptomycin in plates coated with human plasma Fibronectin (100 μg ml^−1^, EMD Millipore). The identity of purified NPCs from ICR and conditional mutant mice was verified by immunostaining using anti-CITED1 (1:200, Cell Signaling), anti-SIX2 (1:200, Proteintech) and anti-LEF1(1:100, Cell Signaling) antibodies, and RT–qPCR analysis of cap mesenchyme and cortical interstitium markers before and after growth factor/inhibitor treatments.

### Transfections and dual-luciferase reporter assays in NPCs

E17.5 NPCs cultured in KSFM (Thermo Fisher Scientific) media with rh-FGF2 (50 ng ml^−1^) were transfected for 24 h using lipofectamine 2000 (Life Technologies)^24^,^25^. Briefly, 1–2 μg of plasmid DNA and 1–2 μl of lipid was mixed in a 1:1 ratio in Opti-MEM (Life Technologies) and added to NPCs in KSFM without antibiotics and incubated for 1 h. Medium was replaced with fresh KSFM supplemented with rh-FGF2 1 h after transfection to minimize cytotoxicity. Transfection efficiency was estimated using a pCX-EGFP construct at 24 and 48 h after transfection and by RT–qPCR for overexpressed genes. For luciferase reporter assays, transfected cells were stimulated with vehicle or indicated growth factors for 24 h. Cells were lysed and luciferase activity was measured using the dual-luciferase reporter assay kit (Promega). Relative luciferase activity was normalized to Renilla-luciferase and average fold changes relative to vehicle treatment from four biological replicates and two independent experiments (*n*=2) are represented in the graphs.

### Immunoblotting

Isolated E17.5 NPCs were treated with rh-BMP7 or rh-FGF9 (50 and 100 ng ml^−1^, R&D systems), TAK1i (0.5 μM, Analyticon Discovery) JNKi (10 μM, Calbiochem) in KSFM (Thermo Fisher Scientific). Total protein was extracted as previously described^7^. Antibodies used were: anti-pTAK1 (Thr184/187), anti-pJNK (Thr183/Tyr185), anti-pJUN (S73), anti-pFOS (S32) (1:1,000, Cell signaling), anti-β-tubulin (1:5,000, Santa Cruz). Protein levels were quantified using FIJI/Image-J software by measuring the integrated density of the indicated proteins normalized to β-Tubulin, the loading control. Average values from two independent experiments (*n*=2) are indicated in the graph.

### Quantitative RT–PCR

RNA extraction from E14.5 and E17.5 NPCs was performed using RNeasy Microkit (Qiagen). Concentration of RNA was measured using NanoDrop2000 Spectrophotometer (Thermo Fisher Scientific), and a final concentration of 100–250 ng μl^−1^ of RNA was used for cDNA synthesis by iScript Reverse Transcription Super Mix (BioRad). Quantitative RT–PCR was performed using iQ-SYBR Green Super mix (BioRad). Primer sequences of genes are listed in Supplementary Table 1. Fold changes were normalized to the housekeeping gene β-actin and average values (mean±s.d.) of three technical replicates and from two to three independent experiments (*n*=2 or 3) are shown in the figures. *P* values were calculated using a two-tailed Student's *t*-test, and *P*<0.05 was considered significant.

### Whole mount immunostaining

Dissected kidneys were fixed in 4% PFA for 10 min at room temperature and washed with 1X PBS for 5 min at 4 °C. Kidneys were permeabilised with 1X PBS containing 0.1% Triton-X for 10 min at 4 °C, followed by a wash in 1X PBS containing 0.01% Tween. Kidneys were incubated with blocking solution containing 1X PBS containing 0.01% Tween with serum of secondary antibody species for 8 h. Primary antibodies to anti-SIX2 (1:200, Proteintech) and anti-cytokeratin8/TROMA-1 (1:100, DSHB) were diluted in blocking solution and added to the wells containing the kidneys and incubated for 24 h at 4 °C. Alexa-Fluor 488/568 secondary antibodies (Molecular Probes) were used at 1:250 and incubated for 24 h to detect staining in the kidneys.

### Cell cycle marker analysis

NPCs were cultured in monolayer with rh-BMP7 and/or rh-FGF9 (50 and 100 ng ml^−1^, R&D systems) in KSFM (Thermo Fisher Scientific) for 24 h. Cells were fixed in 4% PFA and blocked in 1X PBS containing serum of the secondary antibody species, following which they were incubated in primary antibodies to CCNE1 and PCNA (1:200, Santacruz). Alexa-Fluor-488 (CCNE1) and Alexa-Fluor-568 (PCNA) secondary antibodies were used to visualize the staining. Images (5–8) were taken per well for each condition with a minimum of three biological replicates and three independent experiments (*n*=3). Pooled images were analysed by Image-J and number of cells positive for G1 (CCNE1+), G1-S (CCNE1+/PCNA+) and S (PCNA+) phases were counted and divided by the total number of DAPI+ nuclei to determine the percentage of cells representing G1, G1-S or S-phase. Data are represented as percentage of G1 or S-phase cells in each condition.

### EdU labelling of NPCs

E17.5 NPCs were cultured in monolayer with rh-BMP7 and rh-FGF9 (50 and 100 ng ml^−1^, R&D systems) in KSFM. Cultures were incubated with 20 μM EdU (Click-iT EdU Alexa-Fluor 488 Imaging Kit, Life Technologies) 4 h after growth factor stimulation and pulse-chased for 20 h. Fixation, permeabilization and Click-iT reaction was performed according to manufacturer's instructions. Cultures were incubated with anti-pHH3 (1:100, Cell Signaling) antibody for 1 h and Alexa-Fluor-568 secondary antibody was used to visualize the staining. Nuclei were stained with Hoechst 33342 (Life Technologies). Images (5–10)were taken per well for each condition with a minimum of three biological replicates from two independent experiments (*n*=2). Pooled images were analysed by Image-J and number of EdU+ (S-phase) and/or pHH3+ (Mitosis or M-phase) nuclei were counted and divided by the total number of nuclei to determine the percentage of cells in S- and M-phases. Data are represented as percentage of S- or M- phase cells in each condition.

### Morphometrics and statistical analyses

(i) Kidney weight measurements were normalized to body weights to account for any differences in overall body size. Kidney size measurements were determined by calculating the cross-sectional area of the pole-to-pole distance on dissected whole kidneys using SPOT 5.1 imaging software. Measured kidney weights and sizes of individual animals per group are represented in scatter plots. (ii) Quantitation of NPC number and proliferation was performed manually for a minimum of five serial sections 100 μM apart per kidney per genotype and the total number of individual or tamoxifen-treated mice (*n*) analysed per time point is indicated in the figures. Error bars represent mean (s.d.) for each animal per experimental group. (iii) Growth curve analysis was conducted by counting cells using a hemocytometer for a minimum of three to five biological replicates per condition from three independent experiments. Two-tailed Student's *t*-test was performed for all statistical analyses and the resulting *P* values are noted in the figures.

## Additional information

**How to cite this article:** Muthukrishnan, S. D. *et al*. Concurrent BMP7 and FGF9 signalling governs AP-1 function to promote self-renewal of nephron progenitor cells. *Nat. Commun.* 6:10027 doi: 10.1038/ncomms10027 (2015).

## Supplementary Material

Supplementary Information Supplementary Tables 1-7 and Supplementary Table 1
