# Supplementary material for: Concurrent BMP7 and FGF9 signalling governs AP-1 function to promote self-renewal of nephron progenitor cells
Source: Nat Commun. 2015 Dec 4;6:10027. doi: 10.1038/ncomms10027 (PMC4686668; doi:10.1038/ncomms10027)
Supplement: Supplementary Information — Supplementary Tables 1-7 and Supplementary Table 1 [file ncomms10027-s1.pdf]

Supplementary Figures

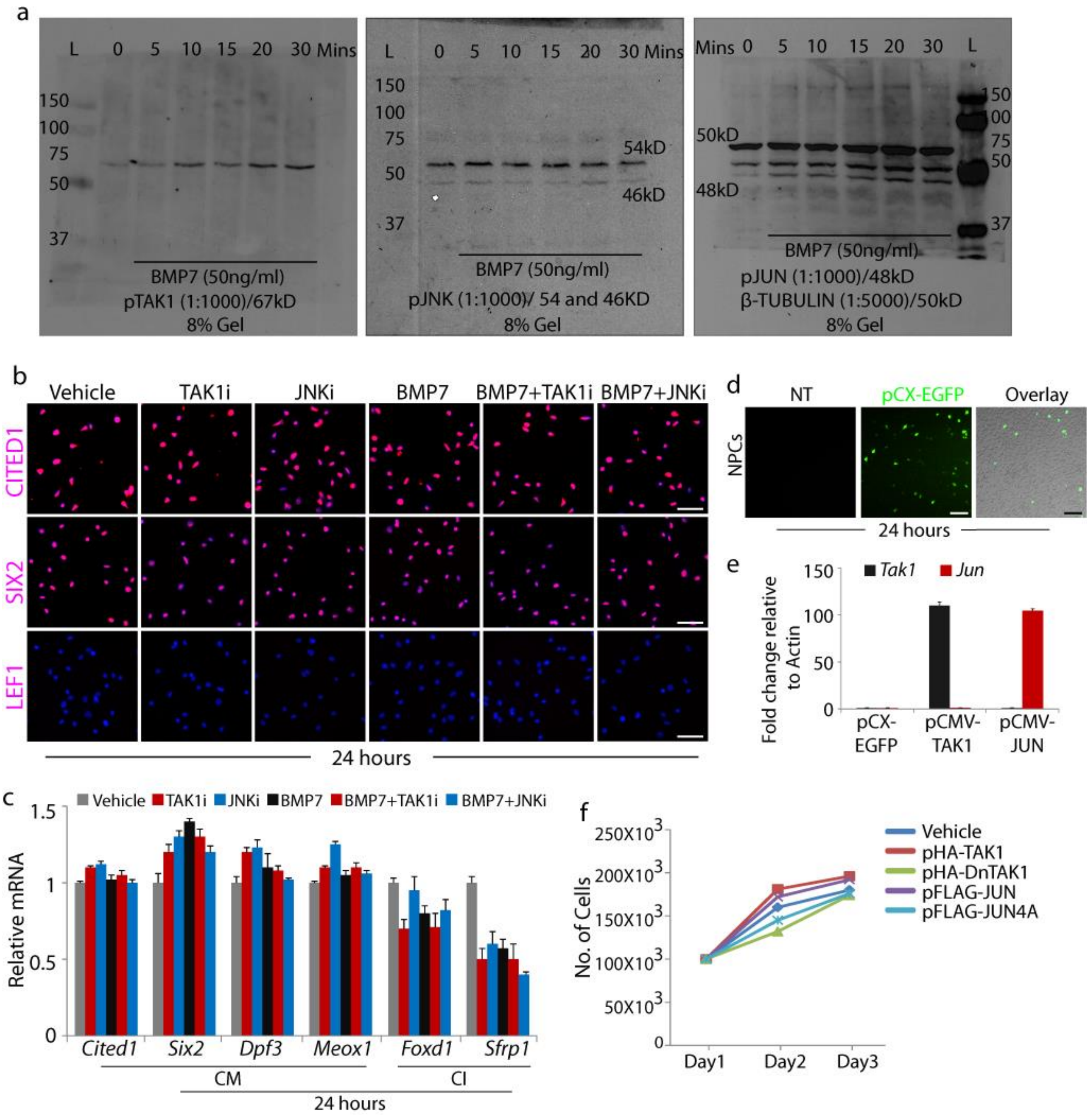

Supplementary Figure 1: Cellular identity of E17.5 nephron progenitor cells

a) Immunoblots of pTAK1, pJNK and pJUN in E17.5 nephron progenitor cells (NPCs) treated with BMP7 in a time course. b) CITED1, SIX2 and LEF1 immunostaining of NPCs treated with vehicle, BMP7, TAK1i and JNKi for 24 hours. Scale bars, 100 μM. c) RT-qPCR of cap

mesenchyme and cortical interstitium markers in NPCs treated with BMP7 and inhibitors of TAK1 and JNK for 24 hours. Error bars indicate s.d. 3 biological replicates per condition, n=3. d) Panels show GFP expression in isolated NPCs transfected with pCX-EGFP construct using lipofectamine for 24 hours. Scale bars, 200 μM. e) RT-qPCR of *Tak1* and *Jun* expression in NPCs transfected with pCX-EGFP, pCMV-TAK1 and pCMV-JUN for 24 hours. Error bars represent s.d. 2 biological replicates per condition, n=2. (f) Growth curve of NPCs transfected with wildtype and mutant constructs of TAK1 and JUN using lipofectamine for 24 hours. Error bars represent s.d. 3 biological replicates per condition, n=3. Abbreviations: CI, cortical interstitium; CM, cap mesenchyme.

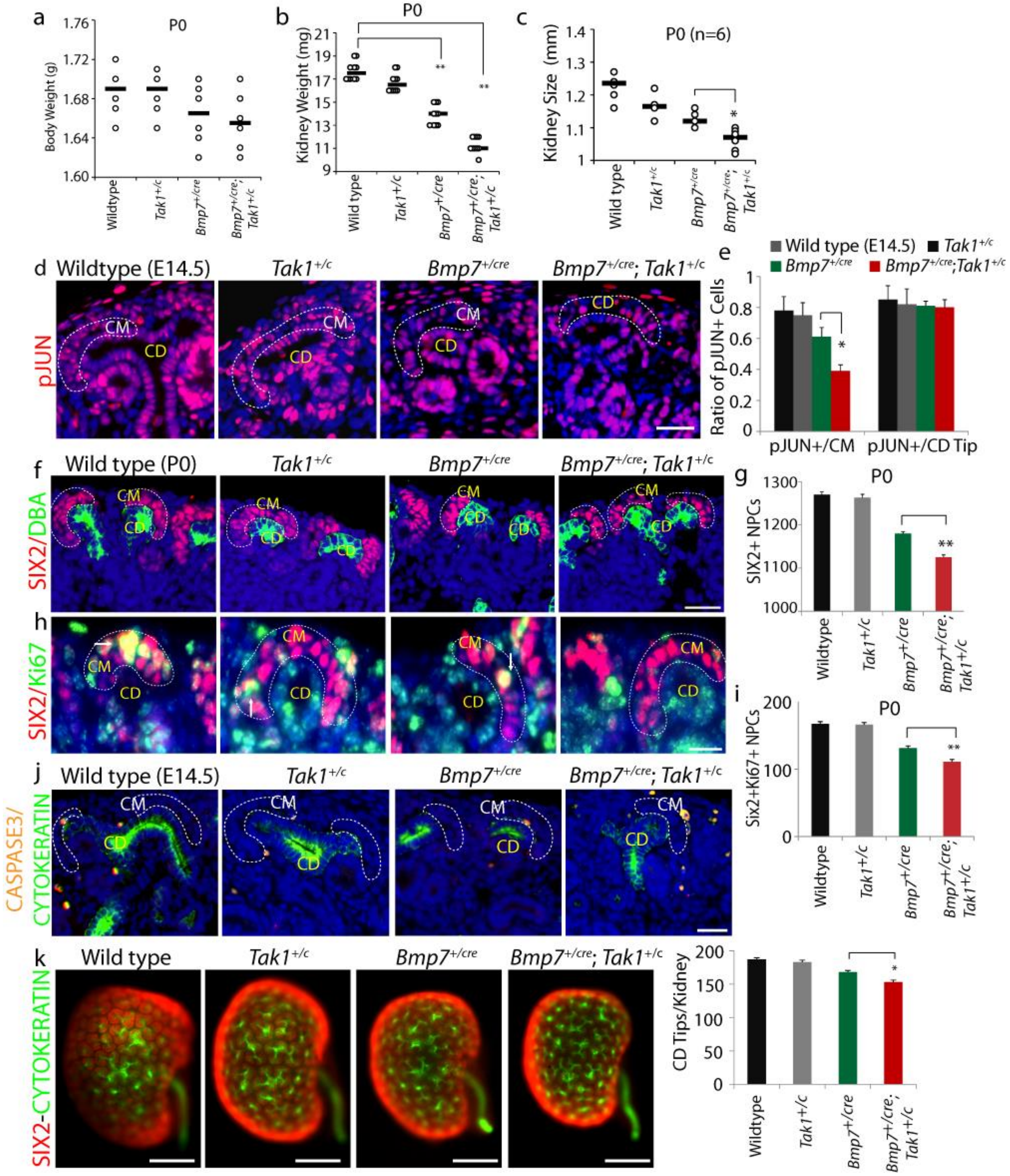

Supplementary Figure 2: *Bmp7* and *Tak1* interact to control NPC renewal

(a-c) Body and kidney weights, and kidney size of P0 wild type, $Tak1^{+/c}$, $Bmp7^{+/cre}$ and $Bmp7^{+/cre};Tak1^{+/c}$ kidneys. ***P*<0.005 and **P*<0.05, Student's t-test. Number of mice analyzed

per genotype (n) is shown on the panel. (d,e) pJUN immunostaining in E14.5 wild type, *Tak1*$^{+/c}$, *Bmp7*$^{+/cre}$ and *Bmp7*$^{+/cre}$*;Tak1*$^{+/c}$ kidneys. Ratio of pJUN+ cells to total number of cells per cap mesenchyme and collecting duct tip is represented in the graph. 10-15 collecting duct tips and cap mesenchymes were analyzed per kidney per genotype. **P*<0.05, Student's t-test. Scale bars, 50 μM. (f-i) Immunostaining for SIX2 (red, cap mesenchyme), DBA Lectin (green, collecting duct) and Ki67 in P0 wild type, *Tak1*$^{+/c}$, *Bmp7*$^{+/cre}$ and *Bmp7*$^{+/cre}$*;Tak1*$^{+/c}$ kidneys. Number of SIX2+ NPCs and Ki67+/SIX2+ cells (white arrows) per kidney section for the indicated genotypes. Error bars indicate s.d. ***P*<0.005, Student's t-test. Scale bars, 100 and 50 μM. (j) CASPASE3 (red, apoptosis marker) and CYTOKERATIN-8 (green, collecting duct) staining in the conditional mutants. Scale bars, 50 μM. (k) Whole mount immunostained images showing SIX2 + NPCs and CYTOKERATIN+ collecting duct tips in the conditional mutants. Number of collecting duct tips scored per kidney per genotype (n=3) is shown in the graph. Error bars represent s.d. **P*<0.05, Student's t-test. Scale bars, 500 μM. Cap mesenchymes highlighted by white dotes lines. Abbreviations: CD, collecting duct; CM, cap mesenchyme.

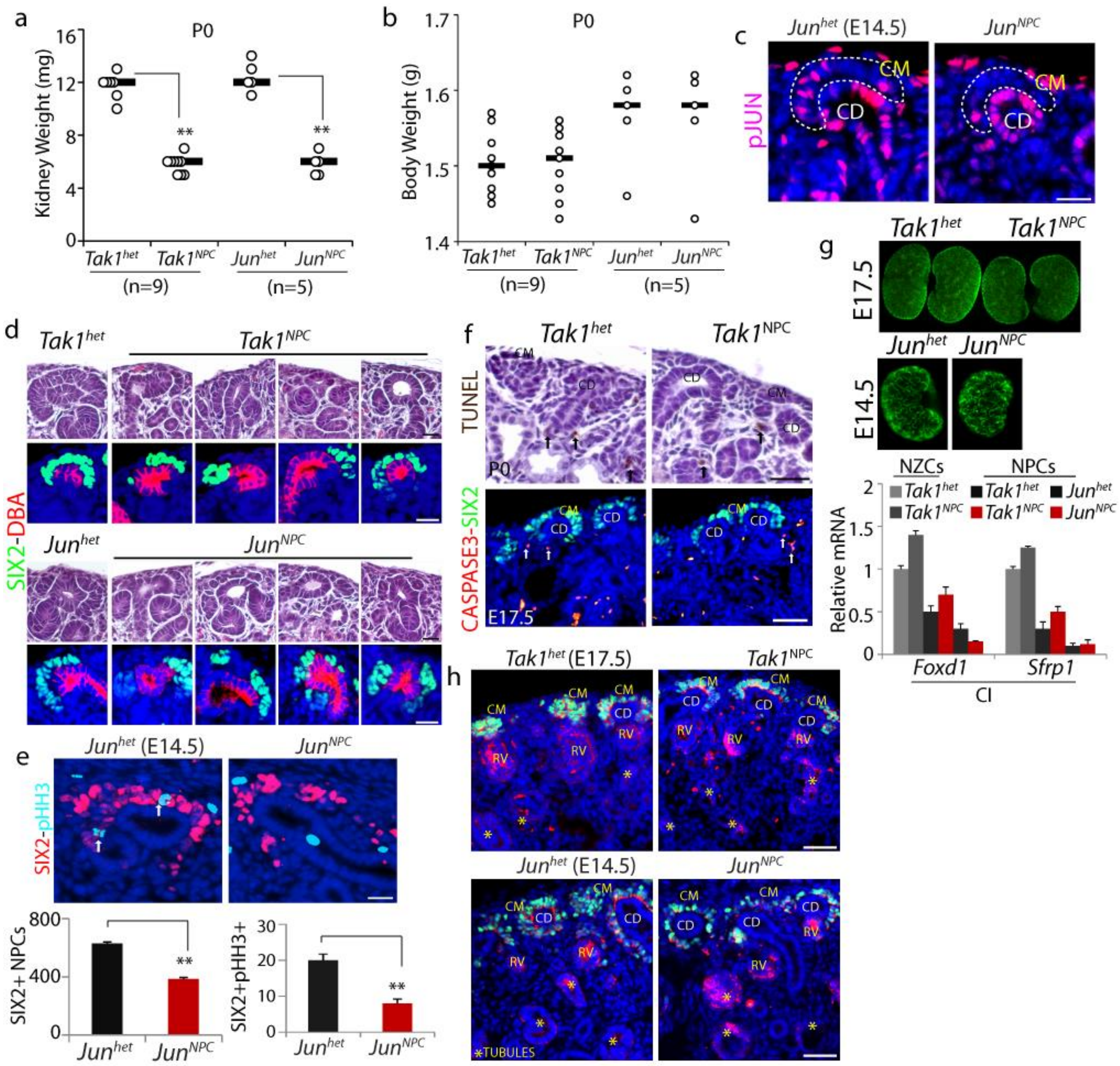

Supplementary Figure 3: *Tak1* and *Jun* are required for self-renewal of NPCs

(a,b) Kidney and body weights of P0 *Tak1*^{het}, *Tak1*^{NPC}, *Jun*^{het} and *Jun*^{NPC} kidneys. Number of mice analyzed per genotype (n) is indicated in the graphs. ***P*<0.005, Student's t-test. (c) pJUN immunostaining in E14.5 *Jun*^{het} and *Jun*^{NPC} kidneys. Cap mesenchyme marked by white dotted lines. Scale bars, 50 µM (d) H&E staining and co-immunostaining of SIX2 (green, cap mesenchyme) and DBA lectin (red, collecting duct) in P0 *Tak1*^{het}, *Tak1*^{NPC}, *Jun*^{het} and *Jun*^{NPC}

kidneys. Scale bars, 25 μM. (e) SIX2 (red, cap mesenchyme) and pHH3 (blue, proliferation marker) co-immunostaining in E14.5 *Jun*^{het} and *Jun*^{NPC} kidneys. Number of SIX2+ NPCs and pHH3+/SIX2+ cells (white arrows) per kidney section is represented in the graph Error bars indicate s.d. $^{**}P<0.005$, Student's t-test. Scale bars, 50 μM. (f) TUNEL (black arrows) and CASPASE3 (red, apoptosis marker/white arrows) and SIX2 (green, cap mesenchyme) immunostaining in P0 and E17.5 *Tak1*^{het} and *Tak1*^{NPC} kidneys. Scale bars, 100 μM (g) E17.5 *Tak1*^{het} and *Tak1*^{NPC} and E14.5 *Jun*^{het} and *Jun*^{NPC} whole kidneys showing GFP fluorescence. RT-qPCR of cortical interstitium markers *Foxd1* and *Sfrp1* in NZCs and NPCs isolated from the indicated genotypes. Error bars represent s.d. 2 biological replicates per group, n=2. (h) β-galactosidase (red) and SIX2 (green) immunostaining in *Jun*^{het} (*Six2*$^{+/cre}$;*Jun*$^{+/c}$;*R26RLacZ*) and *Jun*^{NPC} (*Six2*$^{+/cre}$;*Jun*$^{c/c}$;*R26RLacZ*) E14.5 kidneys. Scale bars, 100 μM. Abbreviations: CD, collecting duct; CI, cortical interstitium; CM, cap mesenchyme; RV, renal vesicle.

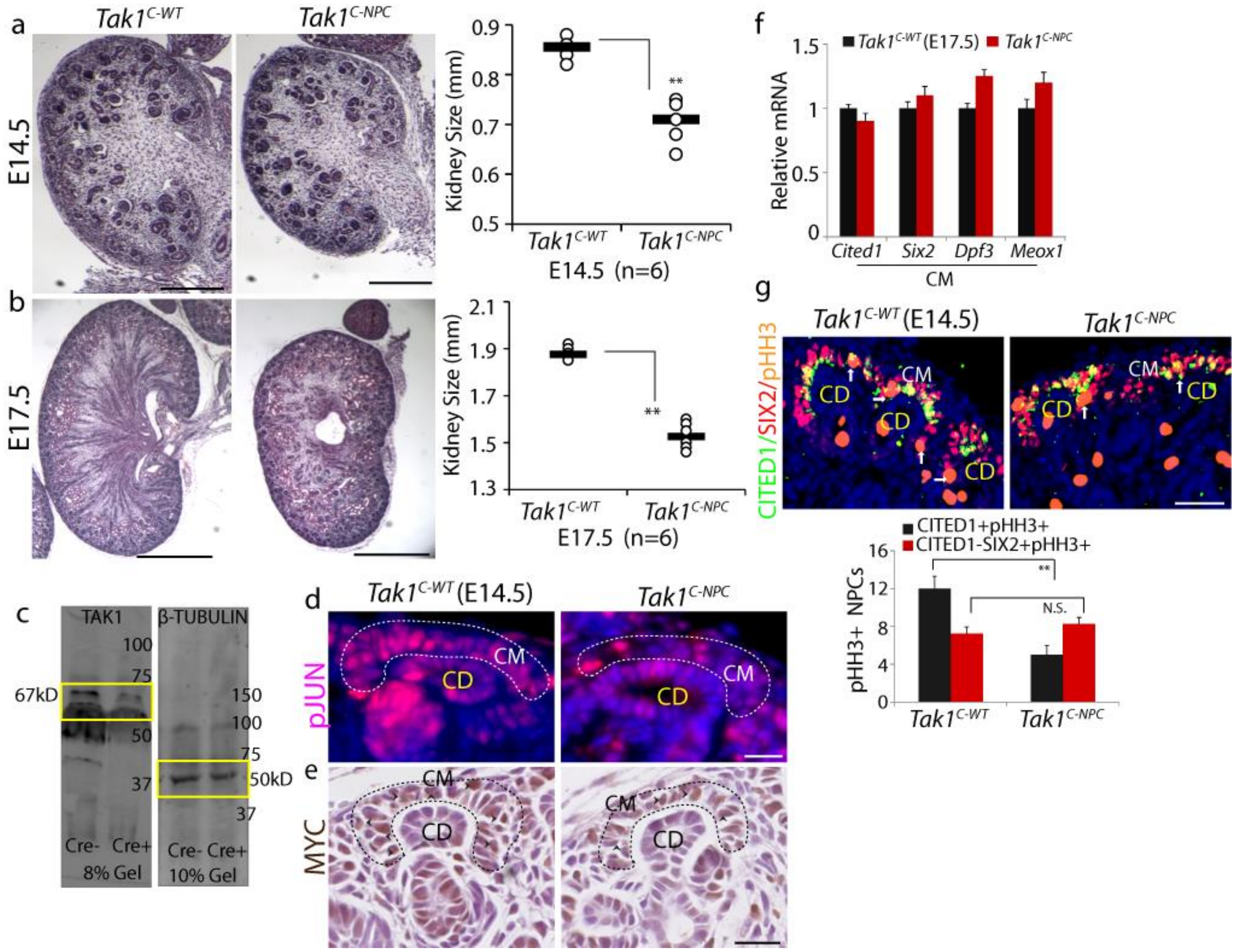

Supplementary Figure 4: Inactivation of *Tak1* in the early CITED1+ SIX2+ NPCs

(a,b) H&E staining of E14.5 and E17.5 $Tak1^{C-WT}$ and $Tak1^{C-NPC}$ kidneys. Kidney size at different stages. Number of mice analyzed per genotype (n) is indicated in the graphs. **P<0.005, Student's t-test. Scale bars, 500 μM and 1 mm. (c) Immunoblots of TAK1 and β-TUBULIN in NPCs isolated from E17.5 $Tak1^{C-WT}$ and $Tak1^{C-NPC}$ kidneys. (d,e) pJUN and MYC immunostaining in E14.5 kidneys. Scale bars, 50 μM. (f) RT-qPCR of cap mesenchyme markers in NPCs isolated from E17.5 kidneys. Error bars indicate s.d. 2 biological replicates per condition, n=2. (g) Immunostaining for CITED1 (green), SIX2 (red), pHH3 (orange) in E14.5 kidneys. Number of CITED1+ and CITED1+/pHH3+ cells and CITED1-/SIX2+/pHH3+ (SIX2+only) cells per kidney section is shown in the graph. Error bars represent s.d. **P<0.005

and N.S. not-significant *P*=0.1, Student's t-test. Abbreviations: CD, collecting duct; CM, cap mesenchyme.

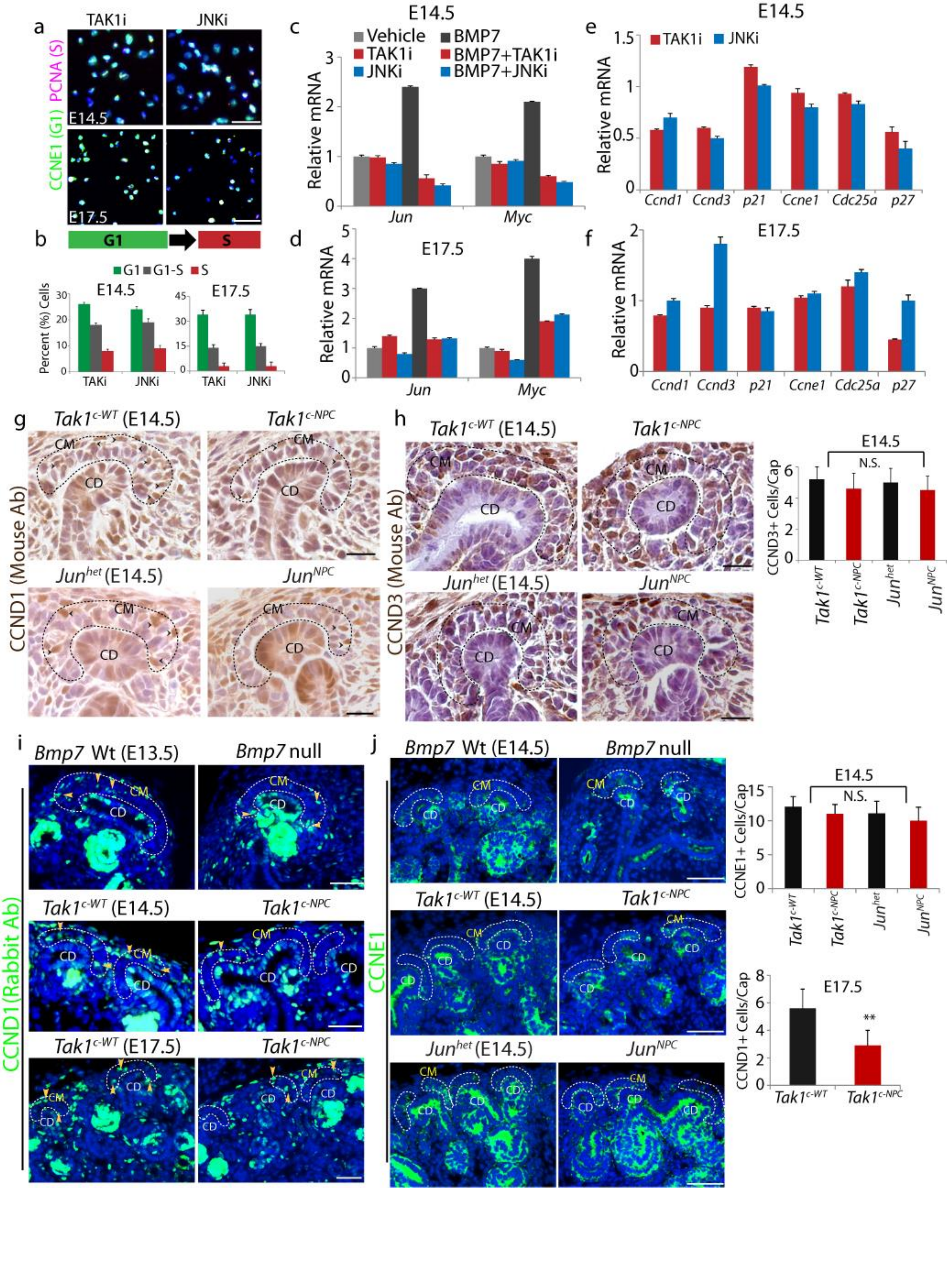
a
TAK1i
JNKi
CCNE1 (G1) PCNA (S)
E14.5
E17.5
b
G1
S
G1 G1-S S
E14.5
E17.5
Percent (%) Cells
TAKi
JNKi
c
E14.5
Vehicle
BMP7
TAK1i
BMP7+TAK1i
JNKi
BMP7+JNKi
Relative mRNA
Jun
Myc
d
E17.5
e
E14.5
TAK1i
JNKi
Ccnd1
Ccnd3
p21
Ccne1
Cdc25a
p27
f
E17.5
g
Tak1c-WT (E14.5)
Tak1c-NPC
Junhet (E14.5)
JunNPC
CCND1 (Mouse Ab)
CM
CD
h
CCND3 (Mouse Ab)
CCND3+ Cells/Cap
N.S.
Junhet
i
Bmp7 Wt (E13.5)
Bmp7 null
Tak1c-WT (E17.5)
CCND1(Rabbit Ab)
j
Bmp7 Wt (E14.5)
CCNE1
CCNE1+ Cells/Cap
CCND1+ Cells/Cap
**

Supplementary Figure 5: BMP7 regulates G1 to S cell cycle progression in NPCs

(a,b) CCNE1 (G phase) and PCNA (S phase) immunostaining in E14.5 and E17.5 NPCs treated with TAK1 and JNK inhibitors. Graph shows percent of G1 and S phase cells in each condition. Error bars indicate s.d. Scale bars, 50 μM. (c,d) RT-qPCR of *Jun* and *Myc* in E14.5 and E17.5 NPCs treated with BMP7 for 2 hours, TAK1 and JNK inhibitors. Error bars indicate s.d. 3 biological replicates, n=3. (e,f) RT-qPCR analysis of cell cycle targets of JUN/AP-1 and MYC in E14.5 and E17.5 NPCs treated with TAK1 and JNK inhibitors. Error bars indicate s.d. 3 biological replicates, n=3. (g,h) CCND1 (mouse antibody) and CCND3 immunohistochemistry in E14.5 $Tak1^{C\text{-}WT}$ and $Tak1^{C\text{-}NPC}$ and Jun^{het}, Jun^{NPC} kidneys. Nuclei are counterstained with hematoxylin. Graphs show number of CCND3+ cells (yellow arrows) scored per cap mesenchyme per kidney section in the indicated genotypes. Erorr bars represent s.d. N.S. indicates not-significant *P*>0.05, Student’s t-test. Scale bars, 50 μM. (i) CCND1 (rabbit antibody) immunostaining in E13.5 *Bmp7* wild type and null, E14.5 and E17.5 $Tak1^{C\text{-}WT}$ and $Tak1^{C\text{-}NPC}$ kidneys. Graphs shows number of CCND1+ cells (yellow arrows) per cap mesenchyme in the indicated genotypes. Error bars indicate s.d. ***P*<0.005, Student’s t-test. Scale bars, 50 and 150 μM. (j) CCNE1 immunostaining in E14.5 *Bmp7* wild type and null, E14.5 $Tak1^{C\text{-}WT}$, $Tak1^{C\text{-}NPC}$, Jun^{het}, Jun^{NPC} kidneys. Number of CCNE1+ cells per cap mesenchyme is represented in the graph. Error bars represent s.d. N.S. indicates not-significant *P*>0.05, Student’s t-test. Scale bars, 150 μM. Cap mesenchymes are highlighted in black or white dotted lines. Abbreviations: CD, collecting duct; CM, cap mesenchyme.

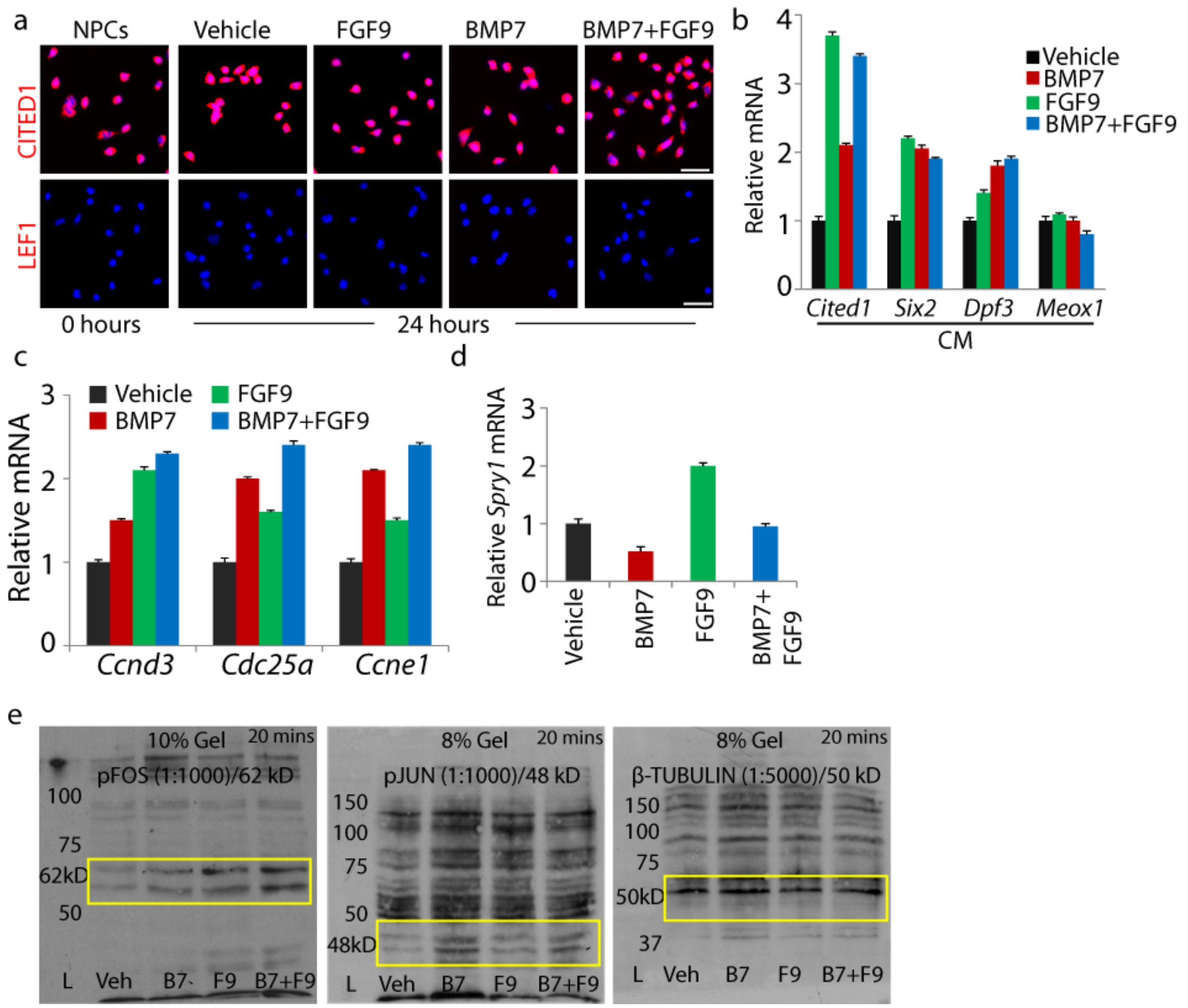

Supplementary Figure 6: BMP7 and FGF9 coordinately regulate AP-1 transcription

(a) CITED1 and LEF1 immunostaining in freshly purified E17.5 NPCs treated with vehicle, FGF9 or BMP7 for 24 hours. Scale bars, 50 μM (b) RT-qPCR of cap mesenchyme markers in NPCs treated with BMP7 and FGF9 for 24 hours. Error bars indicate s.d. 3 biological replicates per condition, n=3. (c,d) RT-qPCR of cell cycle regulatory genes (*Ccnd3*, *Cdc25a*, *Ccne1*) and FGF target gene *Spry1* in NPCs treated with vehicle, BMP7, FGF9 or BMP7+FGF9. Error bars indicate s.d. 3 biological replicates per condition, n=3. e) Immunoblots of pFOS, pJUN and β-TUBULIN in E17.5 NPCs stimulated with Vehicle, FGF9, BMP7 and BMP7+FGF9 for 20 minutes. Abbreviation: CM, Cap mesenchyme.

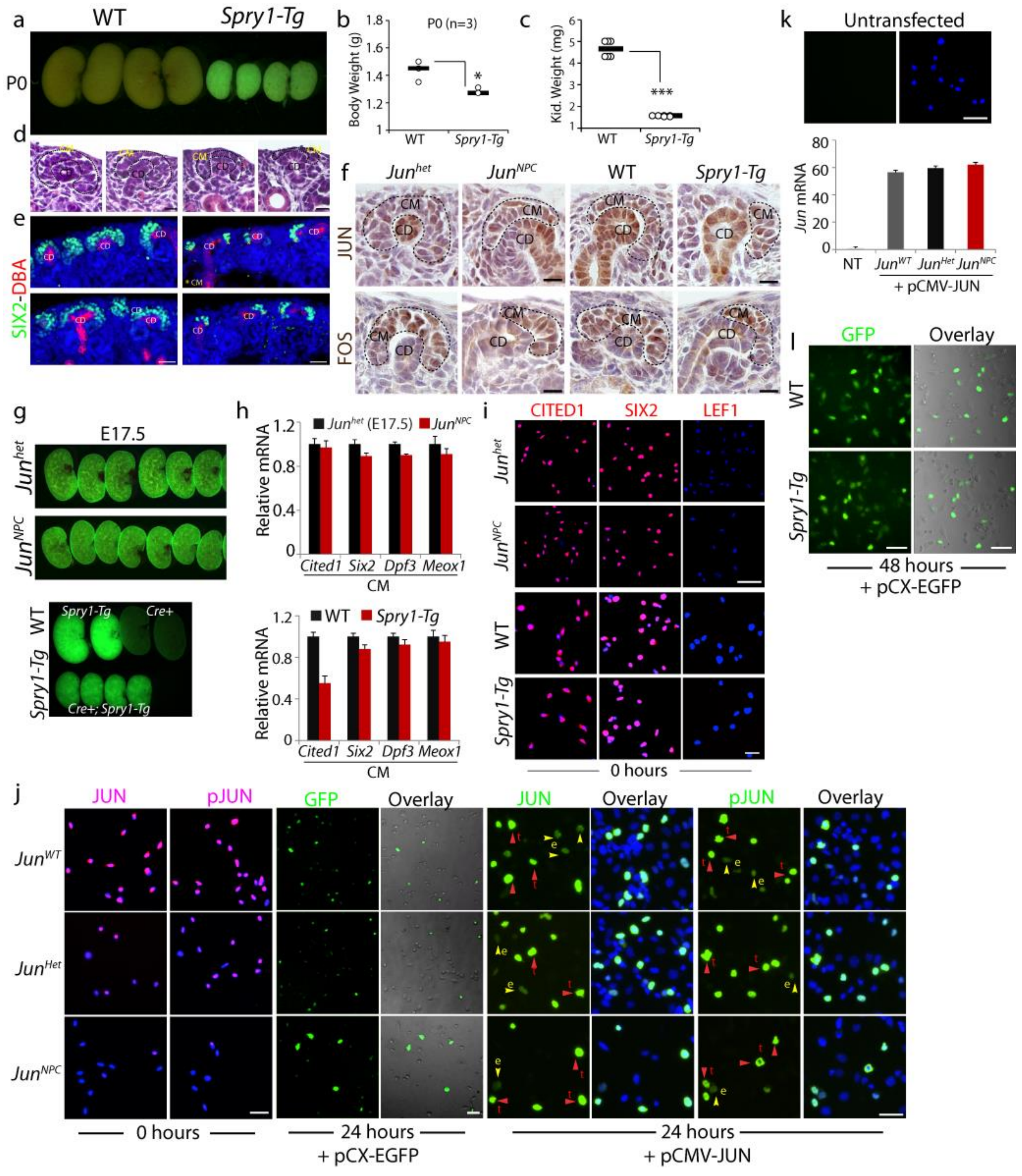

Supplementary Figure 7: Characterization of *Spry1-Tg* kidneys

(a) Stereomicroscope images of P0 wild type (WT) and *Six2-cre*;*Spry1-Tg* (*Spry1-Tg*) kidneys. (b,c) Body and kidney weights of WT and *Spry1-Tg* mice at P0. Number of mice analyzed per genotype (n) is noted in the graphs. **P*<0.05 and ****P*<0.0005, Student's t-test. (d,e) H&E and

co-immunostaining of SIX2 (green, cap mesenchyme) and DBA lectin (red, collecting duct) in P0 WT and *Spry1-Tg* kidneys. Scale bars, 25 and 100 μM. (f) FOS and JUN immunostaining in P0 WT, *Spry1-Tg, Jun*^{het} and *Jun*^{NPC} kidneys. Cap mesenchymes are highlighted in black dashed lines. Scale bars, 50 μM. (g) E17.5 WT, *Spry1-Tg, Jun*^{het} and *Jun*^{NPC} whole kidneys showing GFP fluorescence. (h) Transcriptional analysis of cap mesenchyme markers in E17.5 WT, *Spry1-Tg, Jun*^{het} and *Jun*^{NPC} kidneys. Error bars represent s.d. 2 biological replicates per genotype, n=2. (i) CITED1, SIX2 and LEF1 immunostaining in E17.5 NPCs isolated from WT, *Spry1-Tg, Jun*^{het} and *Jun*^{NPC} kidneys. Scale bars, 50 μM. (j) JUN and pJUN immunostaining in freshly purified NPCs isolated from E17.5 *Jun*^{het} and *Jun*^{NPC} kidneys. NPCs transfected with pCX-EGFP construct showing GFP expression at 24 hours. pJUN and JUN immunostaining in pCMV-JUN transfected NPCs showing endogenous (e, yellow arrows) and over-expressed JUN (t, red arrows) 24 hours after transfection. Scale bars, 50 μM. (k) DAPI staining of un-transfected cells. RT-qPCR of *Jun* expression in NPCs transfected with pCMV-JUN for 24 hours. Error bars represent s.d. 2 biological replicates per condition, n=2. (l) GFP expression at 48 hours in pCX-EGFP transfected E17.5 NPCs isolated from WT and *Spry1-Tg* kidneys. Scale bars, 50 μM. Abbreviations: CD, collecting duct, CM, cap mesenchyme.

Supplementary Table 1

No.	Gene	Primer Sequence (Forward)	Primer Sequence (Reverse)
1	*Actin*	CGTGCGTGACATTAAAGAGAAG	TGGATGCCACAGCATTCCATA
2	*Tak1*	CGGATGAGCCGTTACAGTATC	ACTCCAAGCGTTTAATAGTGTCG
3	*Jun*	CAGTCCAGCAATGGGCACATCA	GGAAGCGTGTTCTGGCTATGCA
4	*Myc*	TCGCTGCTGTCCTCCGAGTCC	GGTTTGCCTCTTCTCCACAGAC
5	*Cited1*	CCACTAGCTCCTCTGGATCG	AGCCCCTTGGTACTGGCTAT
6	*Six2*	CACCTCCACAAGAATGAAAGCG	CTCCGCCTCGATGTAGTGC
7	*Dpf3*	CCTCTCAGGAAGACCACGACAA	CCAGGTGAGTATGAGCGTAGTG
8	*Meox1*	GGAGGATTGCATGGTACTTGGG	CTTTGCTGCTGCCTTCTGGCTT
9	*Foxd1*	CCTACTCGTACATCGCGCTCAT	TAAGGGAAGCGGCTGCTGATGA
10	*Sfrp1*	CAATACCACGGAAGCCTCTAAGC	GCAAACTCGCTTGCACAGAGATG
11	*Ccnd1*	GCAGAAGGAGATTGTGCCATCC	AGGAAGCGGTCCAGGTAGTTCA
12	*Ccnd3*	CGAGCCTCCTACTTCCAGTG	GGACAGGTAGCGATCCAGGT
13	*p21*	TCGCTGTCTTGCACTCTGGTGT	CCAATCTGCGCTTGGAGTGATAG
14	*p27*	TCAAACGTGAGAGTGTCTAACG	CCGGGCCGAAGAGATTTCTG
15	*Cdc25a*	ACAGCAGTCTACAGAGAATGGG	GATGAGGTGAAAGGTGTCTTGG
16	*Ccne1*	GTGGCTCCGACCTTTCAGTC	CACAGTCTTGTCAATCTTGGCA
17	*Fos*	GGGAATGGTGAAGACCGTGTCA	GCAGCCATCTTATTCCGTTCCC
18	*Spry1*	ATGGATTCCCCAAGTCAGCAT	CCTGTCATAGTCTAACCTCTGCC
19	*Pea3*	CACAGACTTCGCCTACGACTCA	GCAGACATCATCTGGGAATGGTC
